# Liquid-phase sequence capture and targeted re-sequencing revealed novel polymorphisms in tomato genes belonging to the MEP carotenoid pathway

**DOI:** 10.1038/s41598-017-06120-3

**Published:** 2017-07-17

**Authors:** Irma Terracciano, Concita Cantarella, Carlo Fasano, Teodoro Cardi, Giuseppe Mennella, Nunzio D’Agostino

**Affiliations:** CREA-OF, Consiglio per la ricerca in agricoltura e l’analisi dell’economia agraria, Centro di ricerca Orticoltura e Florovivaismo, via Cavalleggeri 25, 84098 Pontecagnano Faiano (SA), Italy

## Abstract

Tomato (*Solanum lycopersicum* L.) plants are characterized by having a variety of fruit colours that reflect the composition and accumulation of diverse carotenoids in the berries. Carotenoids are extensively studied for their health-promoting effects and this explains the great attention these pigments received by breeders and researchers worldwide. In this work we applied Agilent’s SureSelect liquid-phase sequence capture and Illumina targeted re-sequencing of 34 tomato genes belonging to the methylerythritol phosphate (MEP) carotenoid pathway on a panel of 48 genotypes which differ for carotenoid content calculated as the sum of β-carotene, *cis*- and *trans*-lycopene. We targeted 230 kb of genomic regions including all exons and regulatory regions and observed ~40% of on-target capture. We found ample genetic variation among all the genotypes under study and generated an extensive catalog of SNPs/InDels located in both genic and regulatory regions. SNPs/InDels were also classified based on genomic location and putative biological effect. With our work we contributed to the identification of allelic variations possibly underpinning a key agronomic trait in tomato. Results from this study can be exploited for the promotion of novel studies on tomato bio-fortification as well as of breeding programs related to carotenoid accumulation in fruits.

## Introduction

In the post-genomics era, the identification of naturally occurring sequence variation in coding as well as regulatory regions of genes is becoming more and more feasible. Sequence capture and target-enrichment methods, followed by high-throughput re-sequencing and allele mining, are ideal tools to address that issue^1–3^. At present, both on-array- or solid-based hybridization and in-solution- or liquid-based hybridization methods^4, 5^ are available to researchers involved in the investigation of genomic regions of interest, being these latter the most widely used. Historically, sequence capture was first applied for targeted re-sequencing of human disease candidate genomic regions and for high-throughput mutation profiling and screening^6, 7^. Given the success of this approach, the method was soon transferred and adopted in plant research. Sequence capture has been demonstrated to be powerful and efficient when applied to different plant species, regardless of the capture method and the sequencing technology used. Indeed, since plant genomes are generally large, complex and rich in repetitive elements, sequence capture approaches appeared to be very suitable and convenient for the investigation of natural variation and the analysis of genetic diversity^8–11^, allowing plant breeders to identify and study the effect of different alleles in economically important crops.

Identifying novel and promising alleles having potential application in crop breeding programs, however, is not a trivial task. First of all, it is necessary to perform a phenotypic screening of the available germplasm in the attempt to uncover potential alleles that are associated with traits of interest. Then, using bioinformatics and molecular tools, alleles responsible for the identified traits must be distinguished and traced in order to be introgressed into elite varieties. As an alternative, the identification of informative sequence variations in hundreds of samples can be used to perform large-scale genome-wide association studies (GWAS) in order to explain much of the heritability of common complex phenotypes and support the study of the molecular basis of agronomical traits in plant^12–14^. Finally, the drop in the costs associated with DNA sequencing makes whole genome re-sequencing experiments affordable. This facilitates Single Nucleotide Polymorphism (SNP) discovery in genetic *loci* responsible for variation in phenotypic traits and allows the identification of intra-specific sequence variation to be exploited for breeding purposes^15–17^.

As far as we know, in solanaceous crops very few capture and target-enrichment experiments have been attempted. Resistance gene enrichment and sequencing approaches (RenSeq) have been successfully carried out in both potato (*Solanum tuberosum*) and tomato^18, 19^ and in-solution-based hybridization method has been applied in highly heterozygous autotetraploid potato^20^. A single target-enrichment experiment dealing with 378 genes that are possible targets for antioxidant metabolism has been published so far on tomato^21^.

Tomato fruits are an important source of compounds with known health-promoting effects that are related to their antioxidant properties^22^. Among these, carotenoids are a class of naturally occurring pigments present in plant photosynthetic tissues having fundamental roles in photo-reception and photo-protection. In addition, they are responsible for the colours of many fruits and flowers and are precursors of vitamin A as well as of plant isoprenoid volatiles and signalling molecules^23^. In plants, carotenoids are mainly synthesized through the methylerythritol phosphate (MEP) pathway that is located into plastids. In *S*. *lycopersicum* such a pathway is highly active during fruit ripening, mainly leading to the accumulation of lycopene and α-/β-carotene. In addition, within *S*. *lycopersicum* species a huge natural genetic variability exists related to accumulation of carotenoid pigments in the fruit^24, 25^. As a consequence, tomato fruits represent a good target for bio-fortification breeding programs^26^. To achieve the goal of the present work, we decided to explore tomato gene space and capture interesting genetic variation affecting genes responsible for carotenoid accumulation in tomato fruits in order to identify superior/beneficial alleles useful in future breeding programs.

## Results

### Genotype selection and candidate gene identification

Data on carotenoid content (i.e. *cis*- and *trans*-lycopene and β-carotene) of ~100 cultivated tomato genotypes, measured in two consecutive years of field trials (2011 and 2012) and published by Ruggieri *et al*.^24^, have been used in order to filter out the list of 48 genotypes that have been selected for capture and targeted-enrichment experiments (see Supplementary Table S1). Cluster analysis based on the elbow method suggested that the number of clusters is 3 (exactly the elbow point; see Supplementary Fig. S1); as a consequence, the population was divided in 3 groups according to the average carotenoid content, namely low- (0.67–36.28 µg/g FW) medium- (65.85–113.22 µg/g FW) and high-CC (113.70–205.01 µg/g FW). Thirteen genotypes were assigned to the low-CC cluster, 20 to the medium-CC cluster and, finally, 15 to the high-CC group (see Supplementary Table S1).

Once genotypes have been selected, next step has been the identification of candidate genes belonging to the MEP carotenoid biosynthetic pathway. The reference catalog published on Tomato Genome Consortium^27^ that includes 46 genes was used together with the available RNA-seq expression profile data in order to filter out 34 candidate genes (see Supplementary Table S2). Most of the selected genes are on chromosomes 1 and 8; by contrast, genes on chromosome 9 are missing.

### Sequence capture, target enrichment and re-sequencing

Using the version SL2.40 of the tomato reference genome, the 2.30 iTAG (international Tomato Annotation Group) annotation and the Agilent’s Sure design software we generated our custom target enrichment design. We intended to capture all exons as well as 5 kb upstream the start codon for all the 34 candidate genes. A total of 13,667 baits of 120 nucleotides in length were designed to target 230.311 kb of regions of interest representing 60.311 kb exon regions and 170 kb putative regulatory regions.

Once baits have been designed they have been used to capture and enrich regions of interest. Agilent’s SureSelectXT target enrichment liquid phase system for Illumina paired-end sequencing was used. For each genotype, we obtained an average of ~2 million reads ranging from 1.10 to 2.35 million. An average of 84% of the total raw reads passed the quality filtering step and remained in pairs (see Supplementary Table S3).

**Figure 1:**
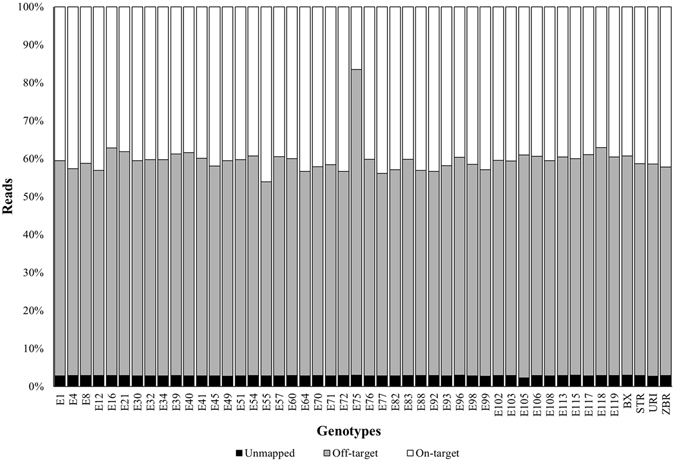
Bowtie2 alignment statistics. Pre-processed reads from the sequencing of 48 tomato genotypes were mapped along the tomato reference genome SL2.40.

Quality-filtered read pairs were aligned along the SL2.40 reference genome using bowtie2. Read-to-genome mapping resulted in ~40% of on-target reads for all the genotypes with the exception of E75 that has been discarded from subsequent analysis (Fig. 1).

**Figure 2:**
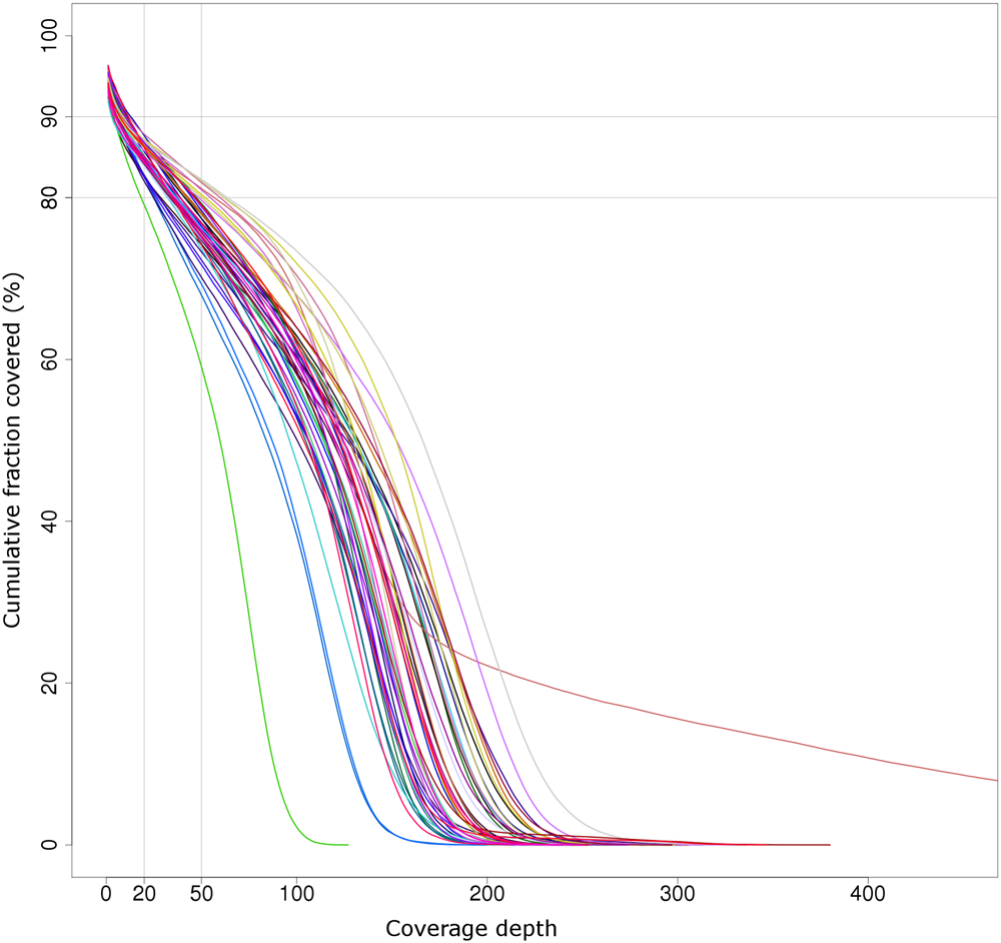
Cumulative distribution of coverage depth across target regions in 47 tomato genotypes. The box in the graph highlights the fraction of bases captured in the target regions covered at a depth between 20x–50x.

**Figure 3:**
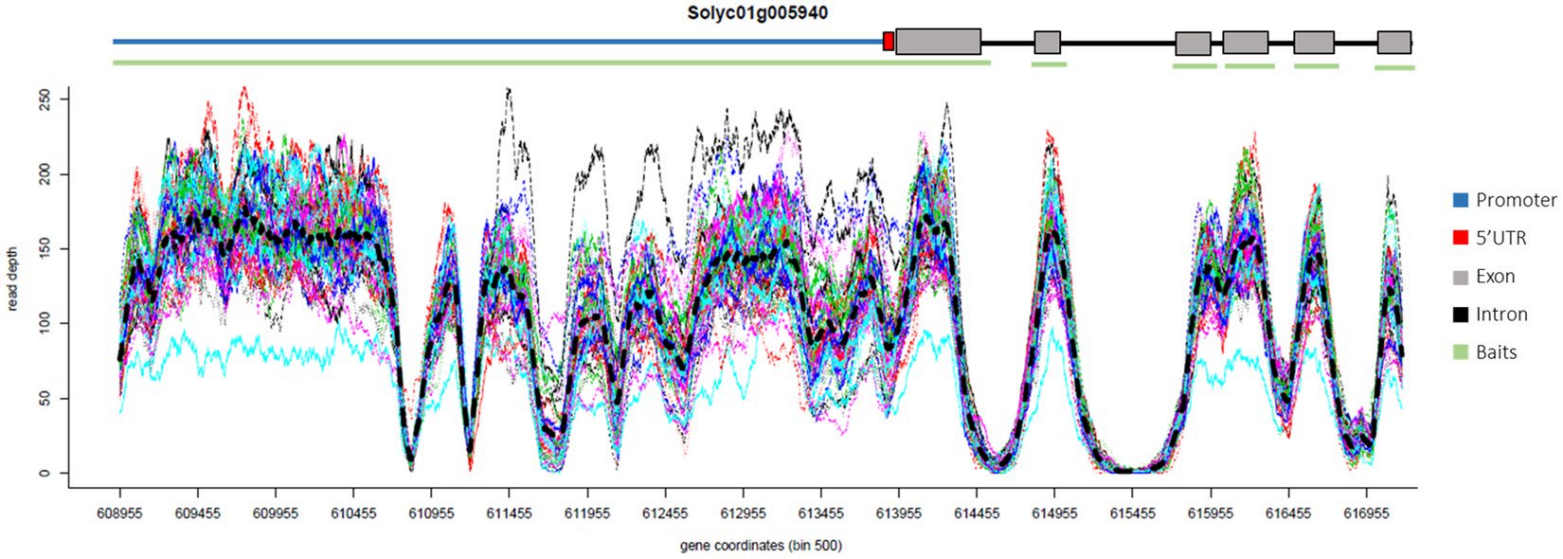
Per-base depth of coverage across gene Solyc01g005940 for the 47 tomato genotypes. Mean value for coverage depth is represented in black bold dashed line. Gene structure and bait positions are shown above the multi-line graph.

On average, more than 80% of bases in the bait regions were covered at a depth ranging from 20x to 50x, while ~8% of bases in target regions were covered at a depth below 20x (Fig. 2). The mean coverage depth estimated for each gene across all 47 genotypes is within a range from 80x to 162x (see Supplementary Table S4). Coverage was sufficiently uniform among genotypes, especially within exon regions. As an example, in Fig. 3 we show the coverage depth for the gene Solyc01g005940 across all the forty-seven genotypes.

### Polymorphism discovery

**Figure 4:**
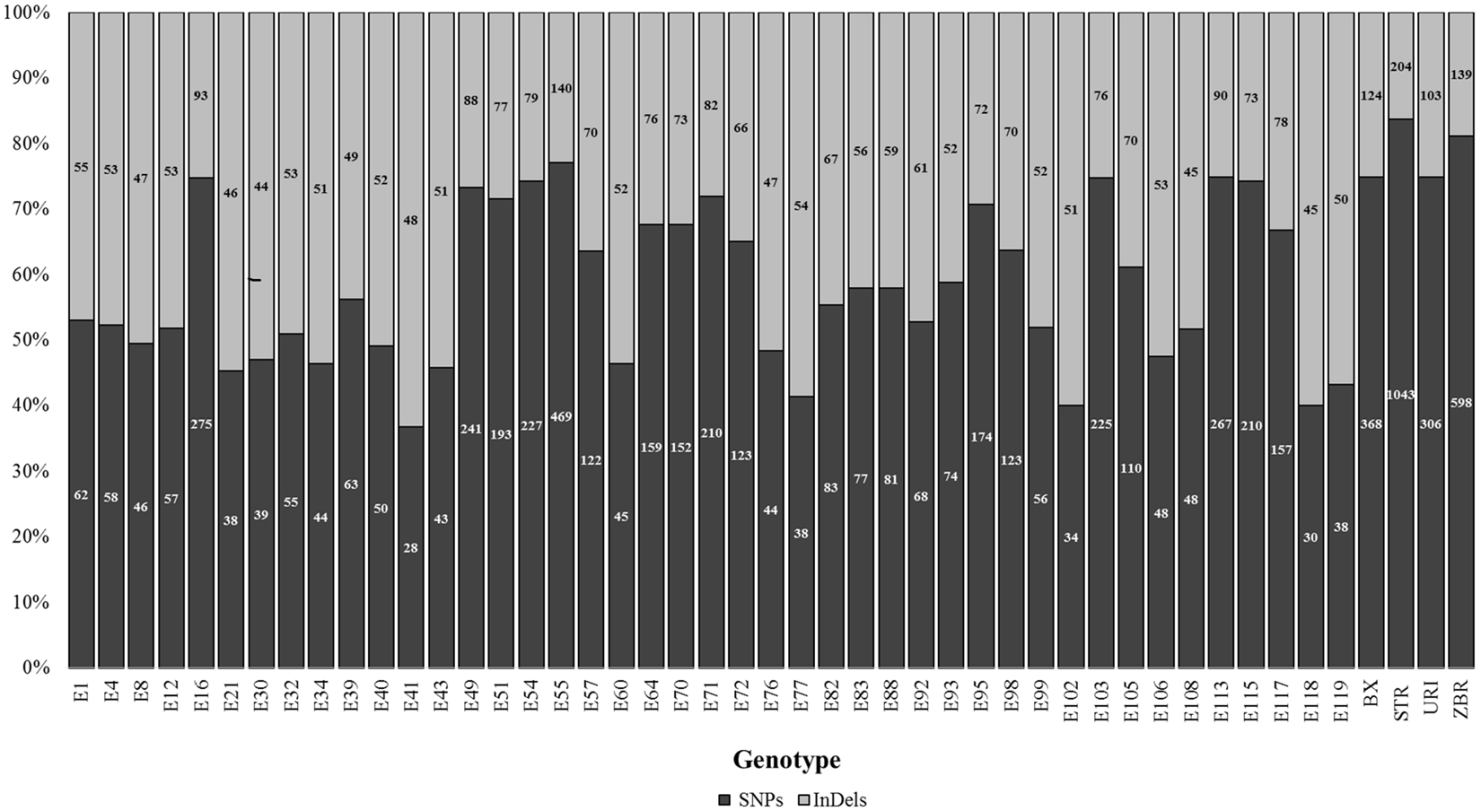
Stacked bar chart showing the number of SNPs and InDels called by GATK across all 47 tomato genotypes under investigation.

Identification of polymorphisms (SNPs and InDels) in high-throughput sequencing data was performed following the Genome Analysis Toolkit best practices workflow^28^ using a minimum Phred-scaled confidence threshold ≥30. A total of 10,263 nucleotide changes (7,080 SNPs and 3,183 InDels) in the target regions has been identified across all 47 genotypes (Fig. 4, see Supplementary Table S5). They correspond to 2,558 non-redundant sequence variations. On avearge ~62% of the identified sequence changes are transitions (Ts), while ~38% are transversions (Tv). The mean Ts/Tv ratio was 1.8 (data not shown). The average frequency of heterozygous polymorphisms across all genotypes was very low (~17%), ranging from 3.9% in Strombolino (STR) to 34% in the genotype E115.

In Supplementary Fig. S2 it is shown a Venn diagram where sequence changes are grouped according to genotype membership within the low-, medium- and high-CC classes. In this way, it is possible to appreciate the number of sequence variation restricted to each class and those shared as a consequence of pair-wise comparisons.

Among all sequence changes called by GATK, 1,155 were the polymorphisms (in more detail 965 SNPs, 112 insertions and 78 deletions) tagged as “private” or genotype-specific. Private polymorphisms affect 41 out of 47 tomato genotypes under investigation, being Strombolino (STR) the genotype that includes the highest number of private sequence changes (335).

For each candidate gene, the reference sequence has been edited in order to insert the identified SNPs/InDels and reconstruct a genotype-specific target genomic region.

Finally, we compared the 2,558 non-redundant sequence changes we found with those recorded in publically available dataset of SNPs and InDels in tomato. Only 90 SNPs and 6 InDels (3.8%) are shared with the dataset published by Shirasawa *et al*.^29^ and Kobayashi *et al*.^30^, respectively. We could not find any overlap between the SNP dataset we generated in this study and the one used for the construction of the tomato SolCAP array^31^.

### Variant annotation and biological effect prediction

Once sequence variation have been identified, we used SnpEff^32^ to classify SNPs/InDels on annotated genes based on genomic location and biological effect. SnpEff also provides a basic evaluation of the putative impact of each variant. As expected, most of the identified variations falls within regulatory regions since only 2,032 (~20%) out of 10,263 mutations we identified are located within gene regions.

**Figure 5:**
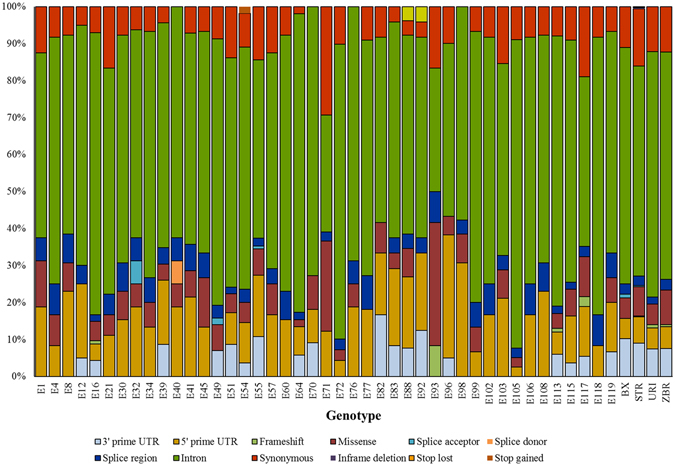
Stacked bar chart showing sequence changes grouped by SnpEff categories within gene regions.

SNPs and InDels affecting gene regions were identified in 30 out of 34 candidate genes (see Supplementary Table S6). The distribution across all 47 genotypes of sequence changes grouped by SnpEff categories is shown in the stacked bar chart reported in Fig. 5. In addition, SNP/InDel patterns can be easily visualized along gene structures (see Supplementary Fig. S3). Among the 671 identified polymorphisms, 73 and 45 affect the 5′ and 3′UTR, respectively; 136 are in the CDS and 417 are located within introns (Table 1). By exploring SNPs restricted to the coding sequences, 79 are synonymous and 52 are non-synonymous SNPs. Among sequence changes limited to introns, only 18 mutations affect splice site sequences at the intron-exon junctions.

**Table 1:** Number and type of sequence changes identified within gene regions.

Gene region	Mutation class	Mutation type	Number
UTRs		5′UTR	73
		3′UTR	45
CDS	Synonymous		79
	Non-synonymous	Missense	52
		Frameshift	2
		In frame deletion	1
		Stop lost	1
		Stop gain	1
Introns		Intron variant	399
		Splice region	16
		Splice acceptor	1
		Splice donor	1

Since sequence polymorphisms in the coding regions could be associated with aberrant protein modifications, all the 52 missense mutations were fed into PredictSNP in order to better predict the effect of amino acid substitutions (Table 2). Nine amino acidic changes were classified as deleterious and 43 as neutral. Genes affected by these 9 missense mutations as well as by other disruptive mutations (i.e. stop gained; frameshift; splice donor; splice acceptor) are highlighted in the schematic representation of the MEP carotenoid pathway depicted in Supplementary Fig. S4. In addition, in order to investigate the possible effect of an in-frame deletion (Thr9del) on the function of the enzyme CCD8 we used PROVEAN. However, the loss of the codon it is predicted to have no influence on protein function.

**Table 2:** List of missense mutations coupled with prediction on the effect of amino acid substitutions by PredictSNP.

Gene	Enzyme	AA Position	Wild residue	Target residue	PredictSNP prediction	PredictSNP confidence	Genotypes		
							Low-CC	Medium-CC	High-CC
*Solyc01g005940*	PSY3	82	C	R	N	0.74	—	—	STR
*Solyc01g090660*	CCD7	337	I	M	**D**	0.72	E98	—	E1, E41, E115
*Solyc01g097810*	ZDS	449	Q	H	N	0.65	—	E55	—
		350	I	T	N	0.83	—	—	STR
		581	L	P	**D**	0.51	—	—	STR
*Solyc02g090890*	ZEP	667	E	G	N	0.83	—	E16, E113	STR, ZBR
*Solyc03g031860*	PSY1	361	V	I	N	0.83	—	E51	—
		105	K	N	N	0.83	E93	—	—
		105	K	M	**D**	0.65	E93	—	—
		108	I	M	N	0.83	E93	—	—
*Solyc03g114340*	DXR	125	T	I	N	0.68	—	—	E96
		56	P	L	N	0.83	—	—	E117
*Solyc04g040190*	LCBY1	405	D	N	N	0.83	E54	E55, E103	E57, E115, STR, ZBR
*Solyc04g050930*	VDE	267	S	N	N	0.74	E54	E55, E103	E57, E115, STR, ZBR
*Solyc04g056390*	GGPPS2	41	Q	K	N	0.83	—	E117	BX, STR, URI, ZBR
		52	V	L	N	0.83	—	E117	URI
		200	S	P	N	0.74	—	E117	BX, STR, URI, ZBR
*Solyc05g010180*	CrtISO_like	142	E	V	N	0.63	—	—	BX
		334	V	I	N	0.83	—	—	BX, STR
		367	K	E	N	0.83	—	—	BX,STR
*Solyc05g016330*	CYP97B2	9	I	N	**D**	0.61	—	E16	—
		17	R	W	**D**	0.55	—	E16	—
*Solyc06g036260*	CHY1	16	F	I	N	0.74	—	—	STR, ZBR
		21	S	T	N	0.83	—	—	STR, ZBR
		27	K	I	**D**	0.55	—	—	STR, ZBR
		122	V	I	N	0.83	—	—	STR, ZBR
		217	A	P	N	0.83	—	—	STR, ZBR
		300	I	K	N	0.83	—	—	STR, ZBR
*Solyc06g074240*	CYCB	20	R	K	N	0.83	E71	—	—
		23	V	F	N	0.63	E71	—	—
		229	R	K	N	0.83	E71	—	—
		289	R	S	N	0.83	E71	—	—
		290	D	N	N	0.83	E71	—	—
		335	V	L	N	0.83	E71	—	—
		473	M	L	N	0.68	E71	—	—
		484	L	V	N	0.83	E71	—	—
*Solyc07g056570*	NCED	424	A	P	N	0.75	—	E51	STR
*Solyc08g016720*	NCED2	571	F	L	**D**	0.87	E88	—	—
*Solyc08g066650*	CCD8	170	V	A	N	0.83	E39, E40, E54, E83, E88, E99	E8, E16, E34, E45, E51, E55, E64, E70, E76, E113, E115	E1, E4, E32, E96, E119
*Solyc08g066720*	CCD_like	267	I	L	**D**	0.51	E21, E71, E82, E93, E98	E103	E30, E96, STR
*Solyc08g075490*	CCD4B	82	E	D	N	0.83	—	E103	—
		272	F	Y	N	0.65	E71	—	—
		295	H	P	N	0.83	—	E55	STR
		486	G	D	N	0.75	—	E55	STR
		488	M	L	N	0.83	—	E55	STR
		501	V	I	N	0.83	—	E55	STR
		518	K	E	N	0.83	—	E55	STR
*Solyc10g081650*	CtrISO	41	I	R	N	0.71	—	E49	STR, ZBR
		362	V	A	**D**	0.76	—	E49	STR, ZBR
*Solyc11g011990*	PTOX	89	D	G	N	0.83	—	E16, E72, E113	E105, URI, ZBR
		106	N	S	N	0.83	—	E16, E72, E113	E105, ZBR
*Solyc11g069380*	HDS	475	I	T	N	0.74	—	E45	—

**N** = Neutral; **D** = Deleterious.

### Identification of cis-acting elements within regulatory regions

The entire regulatory region of candidate genes as defined by 5 kb upstream the translational start site was limited to the intergenic region that spans between the start codon of each candidate gene and the end coordinate of the previous gene. For each candidate gene, reconstructed genotype-specific regulatory region was fed into PLACE db tool that returned as output all the *cis*-acting regulatory elements. A total of 246 *cis*-acting regulatory elements were identified along both the forward and the reverse strand within promoter regions. The smallest number of *cis*-acting elements was found within the regulatory region of the gene Solyc05g10180 (44 *cis*-acting regulatory motifs) while the highest number was detected for the gene Solyc11g011990 (140 *cis*-acting regulatory motifs) (see Supplementary File S1).

**Figure 6:**
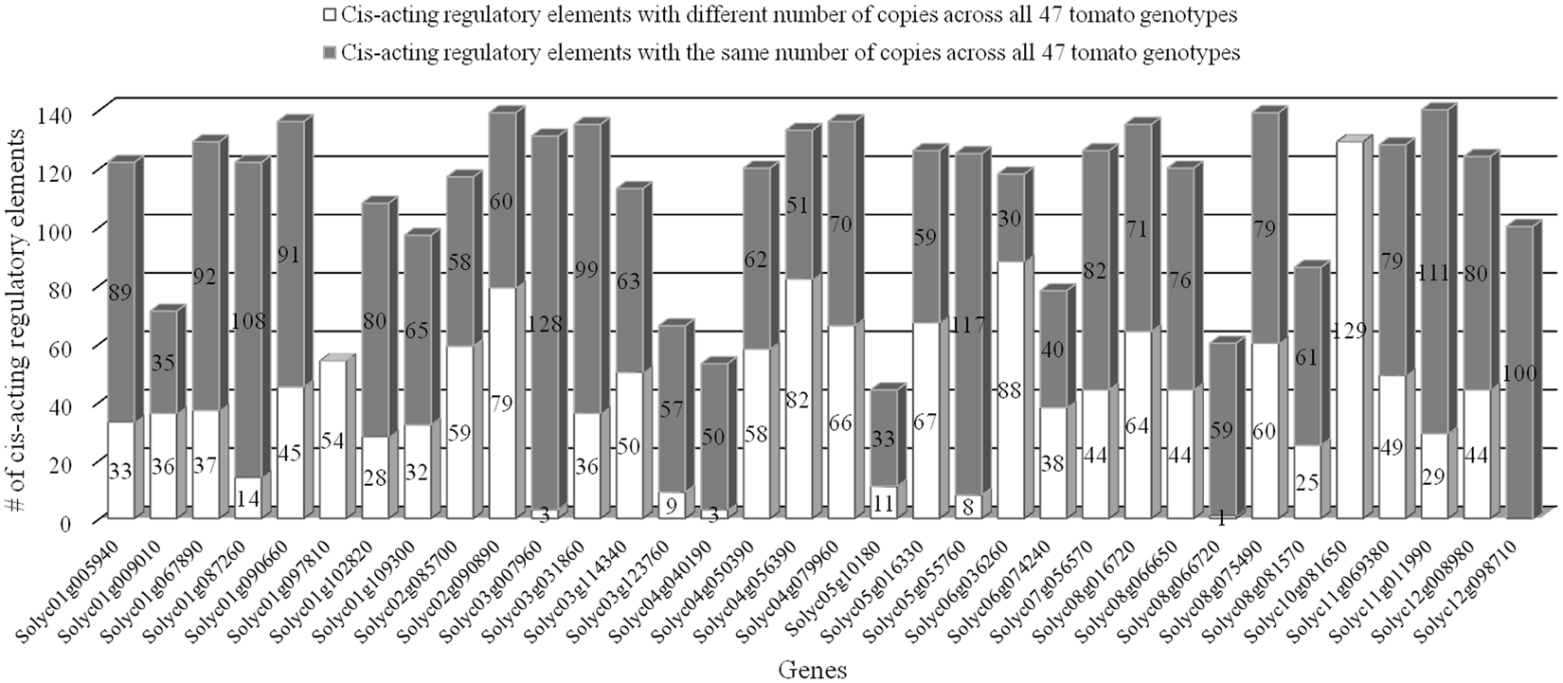
Bar chart showing the copy number variation of *cis*-acting regulatory elements within the promoter region of the 34 candidate genes across all 47 tomato genotypes.

Then, for each candidate gene we reported the number of copies of each *cis*-acting regulatory motif we found in all the 47 tomato genotypes under study. In such a way, we were able to highlight differences in number of *cis*-acting regulatory element copies across genotypes (Fig. 6 and Supplementary File S1). The promoter region of the gene Solyc12g098710 did not show changes in the number of copies of *cis*-acting regulatory motifs, while gene Solyc01g097810 includes several nucleotide polymorphisms that determine 100% variability in the number of all the *cis*-acting regulatory elements we identified (Fig. 6).

### Polymorphism validation by PCR amplifications and Sanger sequencing

A subset of the novel polymorphisms identified by GATK was validated by PCR amplifications and Sanger sequencing. A total of 26 sequence changes, located in both regulatory (10) and gene regions (16) and affecting 16 genes, was selected. Polymorphisms within regulatory regions were chosen randomly, while those within genes were selected among sequence changes responsible for non-synonymous substitutions and splice site variations. PCR amplifications, performed by using 20 primer pairs, resulted in 15 fragments of the expected size. These amplicons allowed a total of 22 polymorphisms to be captured. However, only 11 amplicons (including 17 polymorphisms) were successfully Sanger sequenced in both forward and reverse orientation.

All these mutations (i.e. four SNPs and 2 insertions affecting the regulatory regions as well as 8 SNPs and a single nucleotide deletion within gene regions) were confirmed by sequencing (see Supplementary Table S7). It was not possible to validate two additional polymorphisms within the regulatory region of the gene Solyc03g114340 due to the poor quality of the sequencing. Thus, all the 15 polymorphisms (100%) have additional evidence supporting they are real.

We also performed PCR amplifications on genomic DNA and mRNA isolated from leaves of 6 different genotypes namely Heinz 1706; STR and E12 (belonging to the high-CC class); E40, E83 and E88 (belonging to the low-CC class) in order to prove the retention of the intron II in the genotype E40 which is affected by the T → C point mutation in the splice donor site of exon-intron junction II (see Supplementary Table S6). PCR product of ~450 bp in size in lane 10 (see Supplementary Fig. S5) confirms the retention of the intron.

## Discussion

Allele mining is a powerful strategy to identify allelic variations underpinning key agronomic traits within plant gene pools. However, the discovery of promising alleles having potential application in crop breeding is not a trivial task. Indeed, the availability of sequence-based resources for the target species as well as of reliable and reproducible phenotypic data is crucial for an efficient allele mining activity^33^. Secondly, the application of efficient bioinformatic approaches is essential to accomplish all the tasks required for the extraction of meaningful results: from the analysis of NGS reads, through the identification of sequence polymorphisms, to the annotation of sequence variants^34^.

Traditionally, allele mining studies were mainly focused on the identification of nucleotide variations within coding regions since they may be associated with protein modifications. More recently, it has emerged the need to examine sequence variations within intronic as well as upstream regulatory regions because they may alter gene expression and modify transcription factor binding site patterns, albeit the accurate characterization of such variations is challenging^33^.

In order to contribute to this topic, we apply the Agilent’s SureSelect liquid-phase sequence capture followed by Illumina re-sequencing of genes responsible for carotenoid accumulation in tomato fruits in a panel of 48 genotypes. Recently, a similar effort has been published in tomato by Ruggieri *et al*.^21^ but none of their target genes belongs to the MEP carotenoid pathway. Also these authors have chosen a solution-based capture method but based on the Roche NimbleGen SeqCap EZ target enrichment system. Shigemizu *et al*.^35^ compared the performance of four commercial human whole-exome capture platforms in terms of differences in target region design, target enrichment efficiency, GC bias and variant discovery, but no studies have been yet performed to compare target enrichment solutions on custom designed gene panels both in plants and other organisms.

With slight variations, our data as well as data by Ruggieri *et al*.^21^ suggest that in-solution-based hybridization coupled with Illumina sequencing is a robust and reliable method for the generation of allelic data points.

One of the main parameters to measure the performance of a target enrichment experiment is represented by the specificity that is the percentage of sequences that map to the intended targets^2^. We got a medium level of on-target enrichment efficiency since, on average, the 40% of mappable reads were classified as on-target reads. In order to check whether this result was in line with previously published studies, we reviewed the most recent literature on the topic^8, 21, 36, 37^, and found that the fraction of reads covering the target is quite variable and that this variability depends on the experimental design (*e*.*g*. number of target genes, nature of the target sequences, size of the capture design) as well as on the DNA capture protocol and the sequencing platform used. The nature of target sequences can significantly influence enrichment efficiency. Indeed, our design included the capture of ~230 kb of which approximately 170 kb were represented by putative regulatory regions; as a consequence, the relatively high portion of off-target reads we found can be also ascribed to the presence of vast regulatory regions (up to 5 kb) that generally are rich in GC and may include repetitive elements^38^. Also the size of the capture design affects the efficiency of target enrichment procedure. Indeed, the smaller the capture design, the higher the level of enrichment (https://www.agilent.com/cs/library/brochures/5990-3532en_lo%20CMS.pdf).

For each gene in each data set, we estimated the mean depth of coverage and the coefficient of variation (CV) across genotypes. CV varies from 0.1 to 0.3; this means that coverage was reproducible among genotypes for each target gene. Then, we checked for variability in sequence coverage across regions of interest. As it is evident from Fig. 3, the coverage spectrum is not uniform throughout the target gene: a remarkably uniform coverage across exons can be observed compared to very uneven coverage within the regulatory region.

All considered, since each bait has the potential to capture paralogs and homologus sequences and because it has been demonstrated that enrichment efficiency level can be considerably reduced in upstream regulatory regions^38^, we think our capture was satisfying.

We classified sequence variants into “shared” and “private” based on their distribution in a range of genotypes or in a single accession. As stated in the result section, private polymorphisms affect 41 out of 47 tomato genotypes under study. We also searched for sequence changes specific to genotypes belonging to the low-, medium- and high-CC classes. Indeed, CC-class-specific polymorphisms might be essential in ascribing characteristic phenotypes to a subset of genotypes. Unfortunately, we did not find clear indications on the association between polymorphisms restricted to each class and carotenoid accumulation.

We identified 671 non-redundant sequence changes within gene regions affecting 30 out of 34 candidate genes. When looking at all SNPs in coding regions, 79 are synonymous and 52 are non-synonymous SNPs. The percentage of non-synonymous SNPs is ~42%, a value very close to that obtained from the analysis of an extensive tomato EST collection^39^. Nine out of 52 missense mutations affect 8 genes of the MEP carotenoid pathway and were classified as deleterious (see Supplementary Fig. S4). We uncovered three deleterious point mutations in three enzymes playing a crucial role in carotenogenesis.

We found a private point mutation (A → T) in the phytoene synthase 1 (*psy1*) gene responsible for the K105M substitution in the protein sequence. Such a polymorphism affects only the E93 (White beauty) genotype. However, most likely this mutation has no impact on the functioning of the protein since it is preceded by a stop gained mutation, the effects of which are discussed below.

We observed a private point mutation (T → C) in the ζ-carotene desaturase (*zds*) gene which determines a L581P amino acid substitution only in the Strombolino genotype. Since this mutation falls within the C-terminus of the protein (that is 588 aa in length), we speculate it does not significantly modify the overall 3D structure of the protein. However, Strombolino does not differ particularly from the remaining genotypes within the high-CC class with respect to carotenoid levels.

The carotenoid isomerase (CRTISO) enzyme is affected by the V362A amino acid substitution in 3 different genotypes of which two (STR, ZBR) belong to high-CC class and the remaining one (E49) is part of the medium-CC class. CRTISO activity is to convert tetra-*cis*-lycopene (pro-lycopene) to all-*trans*-lycopene. In tomato it has been described a loss-of-function mutation of *CRTISO* that is the gene that encodes for the *tangerine* locus. Indeed, fruits of *tangerine* mutants are orange and accumulate pro-lycopene instead of all-trans-lycopene^40^. Additional line of evidence of the key role of *CRTISO* in the carotenoid pathway have been reported in literature for melon and Chinese cabbage^41, 42^. The V362A substitution we identified falls right in the middle of the protein (that is 615 aa long), even if tagged as deleterious, would seem not to impair protein function so that all the genotypes harboring this mutation are characterized by having levels of *cis*- and *trans*-lycopene comparable with those of other genotypes in the same CC-class. Knowledge of the effects of the V362A amino acid substitution on protein 3D structure will certainly provide insight into protein mechanism and will support investigations of single mutation effects on carotenoid accumulation.

We revealed also six deleterious point mutations in 5 genes encoding enzymes downstream of β-carotene biosynthesis, namely *chy1*, *cyp97b2*, *nced2*, *ccd7* and *ccd*-*like* (see Table 2). These mutations, although not affecting the content of β-carotene and *trans*- and *cis*-lycopene we quantified via RP-HPLC, could be responsible for a different accumulation of zeaxanthin (one of the most common carotenoid alcohols found in nature), and other carotenoid-derived compounds such as abscissic acid (ABA) and strigolactones. These latter are interesting target in tomato breeding for abiotic and biotic stress tolerance^43, 44^.

In addition to missense mutations, we identified a stop gained and 2 frameshift mutations predicted to have high impact on protein structure and function (see Supplementary Fig. S4). We found a point mutation (C → T) in the first exon of the *psy1* gene that introduces a premature stop codon at position 52 of the protein. Interestingly, this mutation affects 5 genotypes (E54, E83, E88, E92, E93) all belonging to the low-CC class. We also identified a frameshift mutation in the same gene affecting only the E93 (White beauty) genotype which is characterized by having creamy white color fruits. Through TILLING, Gady *et al*.^45^ identified two point mutations in the tomato *psy1* gene. The first is a null mutation (allele knockout) that determines a failure of red coloring of fruits. The second produces an amino acid substitution (P192L) which affects PSY1 activity through mis-folding and results in a delayed accumulation of red pigments in the berry. Similarly to what observed by Gady *et al*.^45^, the stop gained mutation is a null mutation which determines the failure of phytoene synthesis. The residual amount of lycopene and β-carotene we observed in these genotypes can be probably ascribed to *psy2* (Solyc02g081330).

We found a deletion (AT111A) in the *cycb* gene that causes a frameshift mutation in 5 genotypes of which three (STR, URI, ZBR) belong to high-CC and two (E16, E113) to the medium-CC class. Araújo *et al*.^46^ isolated putative alleles of the *cycb* gene from wild and cultivated tomatoes through PCR-based experiments. They found that single base deletions/insertions are fairly common within alleles of the *cycb* gene. Furthermore, Mohan *et al*.^47^ examined a large tomato population (~500 accessions) by EcoTILLING but did find no insertions or deletions other than already described frame shift mutations (A103: and A463ATA). Therefore, the AT111A deletion we identified seems to be unknown and it definitely enriches the panel of allelic variants identified so far for *cycb*.

As is common knowledge, polymorphism rate increases within introns and UTRs. Indeed, about 80 percent of the sequence changes we identified in gene regions falls out from the CDS, more precisely ~62% is located in introns and the remaining ~18% in UTRs. Even though such polymorphisms are more tolerated compared to variations within coding sequences, the presence of mutations in these regions could have effect on mRNA synthesis, stability, translation and accumulation. As expected, introns have a higher InDel rate (65), while slightly less InDels were found within UTRs (28). Indeed, the importance of UTRs in the post-transcriptional regulation of gene expression basically relies on the presence of DNA motifs that can be arranged in hairpins and loops^48^. Such DNA secondary structures play their role interacting with proteins of the transcriptional or translational machinery. That might explain why UTRs tend to accumulate InDels to a lesser extent than introns.

The accurate study on the effects of nucleotide variability within introns and un-translated regions is complicated by lack of efficient automated classification tools and requires extensive experimental demonstrations that are incompatible with high-throughput polymorphism screenings. However, for a particular class of polymorphisms (i.e. that affecting splice site regions), the association with phenotype attributes can be more easily explained.

Our dataset includes 18 mutations located in splice site regions, two of which affect splice site boundaries and seem to have high-impact effects. Precisely, mutations at splice donor or acceptor sites can induce intron retention, exon skipping or can lead to the activation of new cryptic exons. This, in turn, could lead to the production of truncated or abnormal proteins^49^.

We found a point mutation (T → C) in the splice donor site of exon-intron junction II of the *psy1* gene which affects only the E40 genotype belonging to the low-CC class and characterized by having yellow fruits (see Supplementary Fig. S4). We have shown via PCR experiments that mutation determines intron retention (see Supplementary Fig. S5) and that this, in turn, impairs the activity of PSY; as a consequence, tomato fruits show a yellow colour. In this regard, some literature data corroborate our f﻿inding. In addition to the already mentioned work by Gady *et al*.^45^, a further evidence of the key role PSY1 plays in carotenoid biosynthesis derives from observations by Kim *et al*.^50^. The authors have described a point mutation at the splice acceptor site of the fifth intron of the *psy1* gene that causes less pigmentation in orange Habanero pepper fruits. Interestingly, intron V from red Habanero harbours the canonical AG splice acceptor site.

We also found a point mutation (A → T) in the splice acceptor site of exon-intron junction I of the *ipp1* (isopentenyl pyrophosphate isomerase 1) gene (see Supplementary Fig. S4). Such a mutation affects one genotype with high-CC (STR) and three genotypes (BX, E49, E55) with medium-CC. A recently published manuscript describes mutations in a locus termed *fcd1* (FRUIT CAROTENOID DEFICIENT 1) that encodes for the enzyme isopentenyl diphosphate isomerase 1. Such mutations reduce overall carotenoid accumulation in tomato fruits^51^. Given the phenotype we observed (red coloured fruits), that is in contrast to what is reported by Pankratov *et al*.^51^, we assumed that the mutation we found removes the normal AG acceptor site in intron 1 and thus allows the use of alternative acceptor sites. This seems not to affect protein function.

The key role of transcriptional regulation of some biosynthetic genes in controlling carotenoid production and determining specific carotenoid accumulation has been widely demonstrated^52^. Indeed, the analysis of carotenogenic gene regulatory regions is crucial to provide insights into the regulatory basis of carotenoid gene expression during fruit development. However, the study on sequence changes in the upstream regions of genes is challenging since it is still quite complex to identify which variants might be relevant in phenotype definition. For each candidate gene, we scanned up to 5 kb upstream the translational start site to identify transcription factor binding sites (TFBSs) in an attempt to cover the most of sequence changes that can impact gene expression/regulation. Varying number of these elements has been identified in the promoter regions under investigation. It has been described that the number of copies of *cis*-*acting* regulatory elements could have some effects on gene expression and that variation in copy number could also enlarge the distance of TFBSs relative to the transcription start site of a specific gene^53^.

Our data revealed hypothetical novel as well as over- or under-represented TFBSs. However, we were not able to link observed nucleotide variability with different levels of carotenoid accumulation in tomato fruits. Indeed, the meaningful interpretation of TFBS copy number aberration (gain or loss) is complicated by the huge amount of data points as well as by the ambiguous and arbitrary definition of promoter borders (the annotation of promoter regions is missing for tomato). Zooming in on core promoter regions would have allowed to limit the analysis to smaller nucleotide stretches, but their identification is demanding since transcription start site is not annotated for any tomato gene.

In conclusion, we believe the effectiveness of a promoter mining study largely relies on genome annotation accuracy. Even though the tomato genome could be considered a gold-standard reference sequence, we dealt with the first annotation release that needs improvements. Overall, our findings did not allow to draw final conclusions and have further highlighted how less well legible are variations in regulatory regions compared to those within coding regions.

## Conclusion

Tomato plants are characterized by having a variety of fruit colours that reflect the composition and accumulation of diverse carotenoids in the berries. The antioxidant potential of dietary carotenoids is of particular significance in human health and this explains why these tomato pigments received great attention by breeders.

In this work, we applied a targeted enrichment strategy as a rapid and appealing option for high-throughput sequence variant detection. We have produced a reliable probe set to capture genetic diversity within genes belonging to the MEP carotenoid pathway in tomato. We targeted 230 kb of genomic regions and observed ~40% of on-target capture. This is in agreement with the experimental design and with reports from recent literature. Ample genetic variation has been found among all the genotypes under study and an extensive catalog of SNPs/InDels located in both genic and regulatory regions has been constructed and deposited into public repository.

This work has also allowed the genetic basis of some known phenotypes to be identified. Indeed, we demonstrated for the first time that the retention of intron II impairs the activity of PSY1; as a consequence, tomato fruits show a low content of carotenoids and have yellow-coloured barries.

Breeders worldwide could benefit from the accumulation of beneficial alleles from tomato genetic resources. With our work, we contributed to the identification of potentially useful alleles within a relatively small but phenotypically well characterized collection of genotypes. This resource can promote the initiation of new studies on tomato bio-fortification as well as can support investigations on consumers’ attitudes towards novel tomato types (e.g. yellow tomato fruits). Finally, the availability of novel/useful alleles represents a necessary reservoir for tomato improvement via genome engineering based on CRISPR/Cas9 systems^54^. Indeed, knock-in of novel alleles responsible for desired traits into elite genomes (i.e. gene replacement) or, more simply, the precise editing of candidate genes, although still challenging, will be the methods of choice in the next future for crop improvement.

## Methods

### Selection of genotypes and DNA isolation

Forty-eight *S*. *lycopersicum* genotypes, differing in carotenoid content (CC, as sum of *cis*-, *trans*-lycopene and β-carotene), were used in this study (see Supplementary Table S1). These genotypes were filtered out from a panel of ~100 tomato genotypes by exploiting already available data on fruit carotenoid content as determined through reversed phase-high performance liquid chromatography (RP-HPLC) analysis^24^. Plants were grown in sterilized soil and maintained in growth chambers for 5 weeks from sowing at 25 °C with a photoperiod of 16/8 hrs light/dark. Total genomic DNA was extracted from freeze-dried leaves using the DNeasy Plant Mini Kit (Qiagen, http://www.qiagen.com/) following the standard protocol with minor modifications as reported by Zhou and Holliday^8^. DNA quality and integrity was checked by gel electrophoresis, and DNA concentration in each sample was measured using the UV-Vis Spectrophotometer ND - 1000 (NanoDrop Thermo Fisher Scientific, Wilmington, DE, USA) and Qubit® 2.0 Fluorometer (Life Technologies, Carlsbad, CA, USA). A 260/280 ratio of 1.7–2.0 with a minimum concentration of 30 ng/µl was applied as cutoff for acceptable extractions.

### Selection of target genes

We retrieved the reference catalog that includes 46 genes belonging to the carotenoid biosynthetic pathway and, based on the available RNA-seq expression data^27^, we filtered out 34 candidate genes (see Supplementary Table S2). In case of genes encoding for enzyme isoforms we selected those exhibiting the highest and lowest (as negative controls) expression level during all stages of fruit development.

### Probe design

*Solanum lycopersicum* cv. Heinz 1706 chromosome sequences (version 2.40) and their annotation (iTAG 2.30) were downloaded from the SGN ftp server (ftp://ftp.solgenomics.net/tomato_genome) and used in the design of 120 bp baits targeting the exons, the introns (~30 bp downstream to the donor site and upstream to the acceptor site) and the regulatory regions (5 kb upstream the translation start site) of the 34 candidate genes. All the baits were designed using the Agilent’s SureSelect Design software (https://earray.chem.agilent.com/suredesign/) and synthesized by Agilent Technologies (https://www.home.agilent.com/).

### Library preparation, target-enrichment and sequencing

Sequence capture was performed using the SureSelect^XT^ protocol (version 1.6) and the 1 kb-499 kb Custom Kit (Agilent Inc., Santa Clara, CA, USA). Briefly, 3.0 µg of tomato genomic DNA was sheared to an average size of 200 bp via sonication using the Bioruptor NGS (Diagenode, Denville, NJ, USA), followed by end repair, 3′-end adenylation, adaptor ligation, and amplification. The Agencourt AMPure XP beads (Beckman Coulter, Pasadena, CA, USA) were used to purify the libraries following each step. Then, 750 ng of each library were used in-solution-based hybridization capture using biotinylated RNA baits. This step was performed at 65 °C for 24 h on a Mx3005 P™ Real-Time PCR System (Agilent Inc., Santa Clara, CA, USA). After hybridization, target regions were purified using streptavidin-coated magnetic beads and each library was amplified to add index tags. Captured libraries were quantified using the Agilent 2100 Bioanalyzer (Agilent Inc., Santa Clara, CA, USA) and pooled such that each index-tagged sample was present in equimolar amounts in the final sequencing sample pool (at concentration of 2 nM). The pooled samples were subjected to sequencing using an Illumina HiSeq 1500 device in a 2 × 101 paired-end format. Library preparation and sequencing were performed at the Genomix4Life Ltd. (Baronissi, Salerno, Italy).

### Data analysis and variation discovery

Illumina reads (FASTQ, 101 bp paired-end, Phred33) were assessed using FastQC (version 0.10.0; http://www.bioinformatics.babraham.ac.uk/projects/fastqc/). Reads were fed into *fastq_quality_filter* (FASTX-Toolkit) to remove sequences with a quality score lower than 30 in more than 80% of read length. Adapter sequences were trimmed by using Trimmomatic version 0.32^55^. Pre-processed reads were re-paired using the *fastqCombinedPairedEnd*.*py* script (https://github.com/enormandeau/Scripts/blob/master/fastqCombinePairedEnd.py) and aligned to the tomato reference genome (version SL2.40) using Bowtie2 version 2.1.0^56^, with the following parameters: -I (the minimum fragment length for valid paired-end alignments) = 100; -X (the maximum fragment length for valid paired-end alignments) = 400; –N (the number of mismatches permitted per seed) = 1.

BAM files plus the tomato reference genome were loaded into the Integrative Genomic Viewer version 2.3.34 for visualization^57^. The *CoverageBed* tool in the BEDtools package (http://bedtools.readthedocs.org/) was used to calculate the depth of coverage of target regions across each genotype. Depth plots were generated using the ggplot2 in R.

Duplicate reads were identified and removed using the *MarkDuplicates* function in Picard version 1.109, (http://picard.sourceforge.net).

SNPs and InDels were called by the Genome Analysis Toolkit (GATK) version 3.3.0^28^. The GATK pipeline was independently run on each data set following the procedure recommended by the GATK documentation.

Briefly, reads around InDels were realigned and Illumina base quality scores were recalibrated to more closely reflect new mismatch rates. The *UnifiedGenotyper* algorithm was run to call both SNPs and InDels setting *stand_emit_conf* = 30, *stand_call_conf* = 30. All polymorphisms with a Phred-based quality score <20 were tagged as low quality and ignored. Variant calls were produced in form of raw VCF files.

The *VariantRecalibrator* tool was used to separate out the false positive machine artifacts from the true positive genetic variants. One million and 473,798 thousands SNPs from the re-sequencing of six tomato accessions^29^ and 190,656 InDels predicted between Heinz and Micro-Tom genomes^30^ were used as training and truth set in *VariantRecalibrator*. SNPs and InDels along candidate genes were drawn using the FancyGene v1.4 tool^58^.

SnpEff^32^ was used to annotate and predict the effects of variants on genes in order to guide downstream analysis.

PredictSNP^59^ and Provean^60^ were used to predict possible impact of amino acid substitutions/deletions on the structure and function of proteins.

The *vcf2diploid* tool, version 0.2.6a (http://alleleseq.gersteinlab.org/tools.html) was used to reconstruct genotype-specific target genomic.

*Cis*-acting regulatory DNA elements within the putative regulatory regions of the 34 candidate genes were identified using the PLACE db tool^61^.

The NCBI Genome Remapping Service was used to remap polymorphism coordinates from SL2.40 to SL2.50.

### Polymorphism validation by Sanger sequencing

Twenty-seven sequence polymorphisms identified by GATK, namely 8 InDels and 19 SNPs affecting gene as well as regulatory regions, were selected for validation by Sanger sequencing. Primer pairs flanking SNP/InDel sites were designed using Primer3 (http://frodo.wi.mit.edu/primer3/) (see Supplementary Table S7). Genomic DNA from tomato leaves was extracted as described above.

Amplifications were carried out in a final volume of 50-μL including 1 × Phusion HF buffer, 200 μM dNTPs, 0.5 μM of oligos, 100 ng of DNA, and 0.02 U/μL Phusion polymerase (Finnzyme). PCR cycling parameters were: initial denaturation at 98 °C for 30 sec; 7 cycles at 98 °C for 5 sec, 70 °C for 15 sec, and 72 °C for 30 sec; 30 cycles at 98 °C for 5 sec, 60 °C for 15 sec, and 72 °C for 30 sec; final extension at 72 °C for 5 min. After purification of PCR products using AMPure SPRI magnetic beads (Beckman), amplicons were subjected to Sanger sequencing. Electropherograms were carefully inspected by using BioEdit (http://www.mbio.ncsu.edu/BioEdit/bioedit.html).

### Data availability

Illumina raw reads have been deposited at the European Nucleotide Archive (ENA) under PRJEB8566 with accession numbers ERR760714, ERR1802390, from ERR762404 to ERR762438, from ERR1810367 to ERR1810377.

VCF files containing the SNPs and InDels identified for the 47 genotypes have been deposited under the project PRJEB19412.

## Electronic supplementary material

Supplementary Information

Figure S3

Table S5

Table S6

File S1
