# Supplementary material for: Liquid-phase sequence capture and targeted re-sequencing revealed novel polymorphisms in tomato genes belonging to the MEP carotenoid pathway
Source: Sci Rep. 2017 Jul 17;7:5616. doi: 10.1038/s41598-017-06120-3 (PMC5514110; doi:10.1038/s41598-017-06120-3)
Supplement: Supplementary file 1 — Supplementary Information [file 41598_2017_6120_MOESM1_ESM.pdf]

SUPPLEMENTARY TABLES, FIGURES AND FILES FOR:

Liquid-phase sequence capture and targeted re-sequencing revealed novel polymorphisms in tomato genes belonging to the MEP carotenoid pathway.

Irma Terracciano[1], Concita Cantarella[1], Carlo Fasano[1], Teodoro Cardi[1], Giuseppe Mennella[1], Nunzio D'Agostino[1,*]

[1]CREA-OF, Consiglio per la ricerca in agricoltura e l'analisi dell'economia agraria, Centro di ricerca Orticoltura e Florovivaismo, via Cavalleggeri 25, 84098 Pontecagnano Faiano (SA), Italy

[*]Corresponding author: nunzio.dagostino@crea.gov.it

Supplementary Tables

Table S1. List of tomato genotypes, origin, fruit colour and mean carotenoid content (CC), calculated as the sum of *cis*-, *trans*-lycopene and β-carotene, in mature fruits measured in two consecutive growing seasons.

					**cis-lycopene isomers** (µg/g FW)			**trans-lycopene** (µg/g FW)			**β-carotene** (µg/g FW)			**CC** (µg/g FW)		
	**CODE**	**GENOTYPE**	**ORIGIN**	**FRUIT COLOUR**	**Mean**	**SD**	**CV**	**Mean**	**SD**	**CV**	**Mean**	**SD**	**CV**	**Mean**	**SD**	**CV**
**LOW-CC**	E21	Pomodorino Giallo	ITALY	yellow	0.00	0.00	0.00	0.38	0.11	0.29	0.43	0.22	0.53	0.80	0.31	0.39
	E39	Casarbore	ITALY	orange	0.86	0.24	0.28	30.25	15.67	0.52	3.07	1.05	0.34	34.18	15.20	0.44
	E40	GiaGiù	ITALY	yellow	0.09	0.11	1.18	1.45	0.79	0.55	0.42	0.22	0.52	1.96	0.92	0.47
	E54	Latin american cultivar	BOLIVIA	orange	0.00	0.00	0.00	0.62	0.77	1.25	0.27	0.13	0.48	0.88	0.78	0.88
	E71	Caro Red	Unknown	orange	1.15	0.07	0.06	9.79	7.18	0.73	25.14	10.37	0.41	36.08	16.30	0.45
	E75	Gold Nugget	Unknown	orange	0.45	0.52	1.17	13.94	12.12	0.87	2.30	1.16	0.50	16.69	13.46	0.81
	E82	Chang Li	CHINA	yellow	0.00	0.00	0.00	1.76	2.06	1.17	1.21	0.50	0.41	2.97	1.87	0.63
	E83	235	BOLIVIA	yellow	0.02	0.02	1.60	2.95	3.04	1.03	0.60	0.14	0.24	3.56	3.13	0.88
	E88	313	BOLIVIA	yellow	0.10	0.19	1.81	3.12	2.72	0.87	0.46	0.54	1.19	3.67	3.38	0.92
	E92	M-4	BOLIVIA	yellow	0.00	0.00	0.00	0.19	0.26	1.37	0.48	0.21	0.43	0.67	0.24	0.36
	E93	White beauty	USA	cream	0.02	0.04	1.71	0.61	0.46	0.75	0.13	0.09	0.71	0.76	0.39	0.51
	E98	1663	GUATEMALA	red	3.04	1.36	0.45	32.06	15.75	0.49	1.18	0.83	0.71	36.28	15.82	0.44
	E99	Tien-min	CHINA	yellow	0.00	0.00	0.00	0.93	0.32	0.34	0.77	0.29	0.38	1.70	0.37	0.22
**MEDIUM-CC**	E8	Corbarino PC05	ITALY	red	2.85	0.88	0.31	102.41	60.86	0.59	3.64	1.21	0.33	108.91	61.80	0.57
	E16	Nero di Crimea	UKRAINE	brownish	3.19	1.33	0.42	79.14	50.13	0.63	2.23	0.58	0.26	84.56	50.67	0.60
	E34	Sorrento PS04	ITALY	pink	3.27	1.19	0.36	93.04	43.78	0.47	1.52	1.14	0.75	97.84	42.04	0.43
	E43	Principe Borghese	ITALY	red	2.39	1.23	0.51	61.95	24.04	0.39	1.50	0.71	0.47	65.85	24.93	0.38
	E49	Latin american cultivar	PERU'	red	2.96	1.11	0.37	92.09	65.77	0.71	1.14	0.79	0.70	96.19	67.43	0.70
	E51	Latin american cultivar	PERU'	red	2.55	0.97	0.38	82.70	51.41	0.62	1.80	0.67	0.37	87.06	52.74	0.61
	E55	Latin american cultivar	COLOMBIA	red	3.27	1.04	0.32	106.94	43.13	0.40	2.54	1.40	0.55	112.75	44.45	0.39
	E60	Latin american cultivar	PERU'	red	3.44	0.62	0.18	81.06	38.20	0.47	1.68	0.15	0.09	86.18	38.66	0.45
	E64	Cuba Plum	CUBA	red	2.89	0.62	0.21	88.81	22.60	0.25	2.27	0.84	0.37	93.98	23.33	0.25
	E70	Latin american cultivar	PERU'	red	4.21	1.76	0.42	81.41	27.18	0.33	2.10	0.91	0.43	87.72	28.86	0.33
	E72	Moneymaker	Unknown	red	2.56	0.94	0.37	93.53	46.57	0.50	1.03	0.29	0.28	97.12	47.18	0.49
	E76	Black Plum	RUSSIA	brownish	3.85	1.73	0.45	102.44	48.72	0.48	3.92	1.63	0.42	110.21	51.80	0.47

Table S1. (continued)

	CODE	GENOTYPE	ORIGIN	FRUIT COLOUR	cis-lycopene isomers (µg/g FW)			trans-lycopene (µg/g FW)			β-carotene (µg/g FW)			CC (µg/g FW)		
					Mean	SD	CV	Mean	SD	CV	Mean	SD	CV	Mean	SD	CV
MEDIUM-CC	E77	Paul Robeson	RUSSIA	brownish	2.86	0.70	0.24	78.29	40.25	0.51	1.06	0.16	0.16	82.20	40.49	0.49
	E103	Mini Red Current	USA	red	4.02	1.00	0.25	87.19	28.50	0.33	3.75	0.67	0.18	94.95	28.43	0.30
	E106	Cornell 111	USA	orange	4.07	0.74	0.18	105.85	48.14	0.45	3.30	0.55	0.17	113.22	48.27	0.43
	E108	V-L-80	SPAIN	pink	2.01	0.58	0.29	75.91	36.92	0.49	1.45	0.73	0.50	79.38	37.97	0.48
	E113	270	BOLIVIA	red	3.84	1.43	0.37	99.47	54.66	0.55	2.29	0.58	0.25	105.60	55.27	0.52
	E117	Allungato medio grosso	ITALY	red	4.02	1.13	0.28	98.41	48.03	0.49	1.43	0.27	0.19	103.86	47.38	0.46
	E118	Marzano 113	ITALY	red	2.84	1.39	0.49	74.95	40.56	0.54	1.63	1.15	0.70	79.42	42.03	0.53
	BX*	Brixsol	Unknown	red	3.22	1.60	0.50	90.43	38.99	0.43	0.29	0.16	0.56	93.94	39.50	0.42
HIGH-CC	E1	Belmonte PBL01	ITALY	pink	6.53	2.10	0.32	135.31	62.10	0.46	2.15	0.83	0.39	143.99	63.71	0.44
	E4	Canestrino di Lucca	ITALY	red	5.67	2.62	0.46	150.35	70.74	0.47	0.73	0.31	0.42	156.75	73.14	0.47
	E12	Fiaschetto	ITALY	red	5.26	2.40	0.46	139.80	55.65	0.40	4.98	1.14	0.23	150.04	54.03	0.36
	E30	Sel PC07	ITALY	red	4.34	0.60	0.14	153.53	66.83	0.44	3.50	0.74	0.21	161.37	67.11	0.42
	E32	Sorrento PS01	ITALY	pink	8.60	1.22	0.14	180.61	35.48	0.20	3.82	0.57	0.15	193.03	35.49	0.18
	E41	Parmitanella	ITALY	red	5.43	1.00	0.18	136.96	37.99	0.28	2.83	0.34	0.12	145.22	38.64	0.27
	E57	Latin american cultivar	PERU'	red	5.91	1.68	0.28	123.85	39.78	0.32	2.56	0.74	0.29	132.32	40.66	0.31
	E95	1463	EL SALVADOR	red	4.02	1.12	0.28	135.23	73.82	0.55	2.81	0.48	0.17	142.05	74.87	0.53
	E102	Chuco	COLOMBIA	red	5.40	2.15	0.40	174.05	70.80	0.41	2.74	0.77	0.28	182.19	73.34	0.40
	E105	Pera Grande	SOUTH AMERICA	red	5.90	1.75	0.30	162.13	62.27	0.38	2.29	0.42	0.18	170.33	62.65	0.37
	E115	No Known Plant ID	PERU'	red	6.73	1.06	0.16	193.17	56.69	0.29	5.11	2.25	0.44	205.01	58.62	0.29
	E119	Campano	ITALY	red	5.53	1.39	0.25	130.93	59.53	0.45	1.50	0.55	0.36	137.96	61.22	0.44
	STR *	Strombolino	Unknown	red	5.21	1.29	0.25	105.47	18.90	0.18	2.38	1.69	0.71	113.70	20.90	0.18
	URI*	Uriburi	Unknown	orange	2.26	1.37	0.61	112.53	79.19	0.70	1.29	0.71	0.55	116.08	78.97	0.68
	ZBR*	Zebrino	Unknown	brownish	3.79	0.70	0.18	147.34	122.33	0.83	1.23	0.52	0.43	152.36	122.76	0.81

* High-pigment commercial hybrids

Table S2. List of 34 target genes, their coordinates on the tomato reference genome SL2.40 and their expression profiles in the fruits at different developmental stages.

Gene ID	Enzyme	Chromosome	Start[*]	Stop[*]	# exons	1cm fruit[§]	2cm fruit[§]	3cm fruit[§]	MG[§]	B[§]	B10[§]
Solyc01g005940	**PSY3**	1	613955	617183	6	0.0	0.0	0.0	0.0	0.0	0.0
Solyc01g009010	**CMK**	1	3014153	3020442	11	40.1	23.9	20.1	16.5	37.5	54.4
Solyc01g067890	**DXS1**	1	69258969	69263260	10	72.0	85.4	103.2	59.5	176.0	295.4
Solyc01g087260	**CCD1B**	1	73957796	73970716	14	19.6	20.2	29.2	63.9	66.7	85.4
Solyc01g090660	**CCD7**	1	76068751	76072476	7	0.4	0.2	0.1	0.0	0.0	0.0
Solyc01g097810	**ZDS**	1	80276527	80284534	14	31.2	38.0	34.1	32.8	53.2	102.0
Solyc01g102820	**MCT**	1	83279474	83284682	11	33.9	21.6	15.5	10.8	7.1	7.8
Solyc01g109300	**HDR**	1	88049244	88054027	10	119.0	98.7	112.4	102.2	131.4	239.4
Solyc02g085700	**GGPPS3**	2	43100346	43101428	1	35.1	31.9	14.9	20.7	50.2	66.6
Solyc02g090890	**ZEP**	2	46947558	46953158	16	31.8	31.6	35.3	25.4	21.7	45.7
Solyc03g007960	**CHY2**	3	2447872	2450289	7	3.6	7.2	12.3	9.9	20.1	36.9
Solyc03g031860	**PSY1**	3	8606368	8610361	6	24.7	60.1	90.1	188.5	2466.5	3813.6
Solyc03g114340	**DXR**	3	58400724	58406203	12	58.2	50.3	35.0	35.9	35.0	49.2
Solyc03g123760	**PDS**	3	64554061	64561664	16	25.0	25.4	24.3	30.0	57.7	101.4
Solyc04g040190	**LCYB1**	4	31103654	31105156	1	12.5	19.2	8.5	8.6	2.3	6.2
Solyc04g050930	**VDE**	4	48132067	48135623	6	6.9	9.5	5.8	7.0	4.1	2.5
Solyc04g056390	**IPP1**	4	53311908	53315677	7	266.3	130.6	135.6	98.0	124.5	216.3
Solyc04g079960	**GGPPS2**	4	61870171	61871262	1	7.0	4.9	1.5	3.4	41.8	78.2
Solyc05g010180	**CrtISO-like**	5	4377503	4382261	10	6.1	7.6	5.5	12.2	8.1	11.8
Solyc05g016330	**CYP97B2**	5	15575138	15583910	14	27.9	24.6	21.0	19.0	16.4	15.2
Solyc05g055760	**IPP2**	5	64415817	64419127	6	54.9	39.8	21.0	38.4	23.4	6.4
Solyc06g036260	**CHY1**	6	22432178	22434113	7	10.3	9.6	6.3	17.7	11.6	51.3
Solyc06g074240	**CYCB**	6	42288127	42289623	1	1.7	2.5	0.9	2.1	1.1	1.6

Table S2. (continued)

Gene ID	Enzyme	Chromosome	Start[*]	Stop[*]	# exons	1cm fruit[§]	2cm fruit[§]	3cm fruit[§]	MG[§]	B[§]	B10[§]
Solyc07g056570	**NCED**	7	61684846	61686663	1	32.9	83.1	82.1	119.7	118.1	126.5
Solyc08g016720	**NCED2**	8	8634393	8636138	1	20.2	59.2	31.7	14.8	1.4	1.1
Solyc08g066650	**CCD8**	8	52620195	52623269	6	0.7	0.2	0.1	0.0	0.0	0.0
Solyc08g066720	**CCD-like**	8	52751808	52753766	9	0.0	0.2	0.2	0.3	0.0	0.0
Solyc08g075490	**CCD4B**	8	56809898	56812517	2	17.7	16.3	30.2	18.0	0.8	0.0
Solyc08g081570	**GCPE**	8	61751474	61756267	3	27.7	33.0	29.3	23.6	29.5	29.9
Solyc10g081650	**CrtISO**	10	62006972	62011520	13	10.7	13.7	18.0	35.1	98.7	190.9
Solyc11g011990	**PTOX**	11	4937989	4942674	9	14.9	15.8	14.0	25.7	57.3	86.2
Solyc11g069380	**HDS**	11	51063324	51069419	19	133.6	121.8	83.2	89.4	128.0	193.7
Solyc12g008980	**LCYE**	12	2285372	2290327	10	16.2	13.2	9.8	2.2	0.4	0.1
Solyc12g098710	**ZISO**	12	64466750	64470757	4	14.9	17.4	10.9	20.4	797.3	1943.1

[*]Gene coordinates as determined by the international Tomato Annotation Group (iTAG)

[§]RPKM mean value of two replicates as calculated from available RNA-seq data (Tomato Genome Consortium, 2012)

MG=Mature green, B=Breaker and B10=Ten days post breaker

Table S3. Number of input reads obtained from Illumina paired-end sequencing and resulting reads after the pre-processing step.

DNA sample	# input reads	Paired	Un-paired
**BX**	2286876	1852885	411944
**E1**	1952223	1686954	262381
**E102**	1940162	1579585	346680
**E103**	2136507	1827985	302851
**E105**	2052414	1664119	368281
**E106**	1960525	1577374	361686
**E108**	2206385	1832595	363705
**E113**	1931628	1608624	314984
**E115**	1807509	1474219	322415
**E117**	1994993	1626789	350511
**E118**	1724681	1457641	263293
**E119**	1549485	1325356	221126
**E12**	1979826	1714469	263020
**E16**	2228097	1846926	370113
**E21**	1867305	1581795	280375
**E30**	2132129	1781724	345308
**E32**	1881621	1611310	268058
**E34**	1837700	1574668	260611
**E39**	1102641	937392	162226
**E4**	1917342	1657822	260810
**E40**	1961899	1577985	362947
**E41**	1967346	1606996	347139
**E43**	1917848	1605355	308507
**E49**	2112239	1762970	340077
**E51**	2286281	1894913	381218
**E54**	2206672	1825361	370033
**E55**	2241791	1912561	325351
**E57**	2351405	1967244	374247
**E60**	2115070	1774688	332305
**E64**	2324120	1963534	354065
**E70**	2161311	1819044	333194
**E71**	2025904	1915160	360069
**E72**	2107535	1785990	316153
**E75**	2000896	1664951	323795
**E76**	2172346	1779756	377300
**E77**	2315821	1935508	365518
**E8**	1948252	1678818	266607
**E82**	1758136	1529309	227979
**E83**	1999442	1740116	257921
**E88**	1784398	1551292	230996
**E92**	1814162	1572285	239507

Table S3. (continued)

DNA sample	# input reads	Paired	Un-paired
**E93**	1748018	1507683	238165
**E95**	2033877	1663946	353150
**E98**	2081156	1703566	361699
**E99**	2041224	1618102	398248
**STR**	1844052	1512087	316700
**URI**	2126043	1745700	367265
**ZBR**	1977472	1623357	337843

Table S4. Mean and median coverage depth estimations for each candidate gene across all the 47 tomato genotypes.

Gene ID	Mean	Median	SD	CV	BX	E1	E4	E8	E12	E16	E21	E30	E32	E34	E39	E40	E41
Solyc01g005940	104.4	104.1	14.7	0.1	122.2	105.6	97.2	111.7	111.5	103.8	99.3	117.6	95.3	94.7	50.8	94.3	98.4
Solyc01g009010	80.2	79.2	15.2	0.2	87.7	79.5	73.6	79.7	79.2	78.7	64.2	80.4	69.4	67.9	39.4	70.8	75.3
Solyc01g067890	102.4	101.8	21.9	0.2	109.5	104.5	97.5	101.7	100.4	96.4	73.0	96.2	87.7	81.9	49.8	89.6	92.4
Solyc01g087260	102.4	100.1	14.6	0.1	119.7	103.9	96.4	107.2	108.6	106.2	94.7	123.3	89.7	97.9	52.9	93.7	99.7
Solyc01g090660	107.4	107.3	18.8	0.2	121.0	107.1	97.7	110.0	113.1	103.4	90.6	118.0	92.3	94.8	54.5	92.2	103.4
Solyc01g097810	110.9	111.4	16.8	0.2	126.5	112.4	102.6	111.9	122.6	109.1	96.2	125.6	96.8	97.3	54.3	101.1	102.5
Solyc01g102820	105.7	104.4	20.0	0.2	117.8	107.8	96.5	103.8	112.8	105.4	88.7	112.4	90.3	92.2	54.3	92.9	98.2
Solyc01g109300	97.1	94.4	27.2	0.3	100.7	97.7	88.6	86.3	98.7	91.7	74.7	92.7	81.7	82.3	47.8	85.0	91.3
Solyc02g085700	135.7	133.4	20.2	0.1	149.9	137.1	129.5	132.2	148.8	133.4	117.6	154.6	117.0	128.9	68.4	122.3	131.6
Solyc02g090890	91.2	86.8	31.9	0.3	93.5	92.4	85.4	85.3	89.0	83.4	64.2	80.3	70.6	70.7	43.9	75.8	79.8
Solyc03g007960	104.2	100.2	25.4	0.2	103.3	97.9	94.0	93.9	99.8	94.8	140.7	100.5	80.8	81.7	48.9	83.3	92.7
Solyc03g031860	115.5	115.6	15.5	0.1	137.0	117.1	111.0	119.4	123.8	119.1	104.7	134.4	106.8	105.3	59.6	107.2	110.1
Solyc03g114340	106.1	102.2	28.1	0.3	115.7	105.9	96.3	107.3	101.7	93.6	75.8	94.1	82.5	76.8	47.0	94.9	95.8
Solyc03g123760	108.9	109.8	18.7	0.2	118.8	109.8	100.3	113.9	112.5	108.1	92.8	121.7	90.8	95.5	54.8	99.6	102.8
Solyc04g040190	97.8	96.4	15.9	0.2	109.5	102.2	96.4	96.5	95.0	94.7	80.3	99.4	90.7	85.7	46.3	87.4	90.1
Solyc04g050930	88.5	85.9	23.9	0.3	90.1	89.6	79.4	92.3	85.2	79.7	68.9	82.1	71.0	67.3	40.3	77.0	77.0
Solyc04g056390	162.1	158.9	23.0	0.1	187.0	170.5	153.7	180.6	175.3	169.3	151.3	193.1	154.5	146.5	79.4	143.9	156.0
Solyc04g079960	111.0	107.5	34.2	0.3	115.0	115.5	103.6	103.0	108.4	100.4	80.1	104.4	87.3	87.5	52.2	93.3	97.8
Solyc05g010180	88.9	88.3	13.9	0.2	103.8	92.1	82.0	95.8	90.6	83.5	78.3	96.6	84.1	77.3	40.5	82.2	86.1
Solyc05g016330	98.9	97.5	24.3	0.2	104.1	100.5	90.1	93.6	98.6	94.3	74.2	99.6	81.2	84.0	48.9	86.2	91.7
Solyc05g055760	104.2	103.3	19.5	0.2	110.4	105.0	97.7	95.9	108.5	103.3	85.7	112.0	90.0	92.8	52.1	92.8	102.2
Solyc06g036260	84.5	85.6	15.7	0.2	93.2	88.9	79.7	90.0	89.1	83.9	74.0	90.9	76.3	73.0	39.0	76.4	81.1
Solyc06g074240	157.4	156.5	23.0	0.1	188.5	159.4	144.3	171.3	176.1	165.5	146.9	189.8	141.2	147.6	80.3	144.1	156.5
Solyc07g056570	133.7	131.2	39.3	0.3	136.5	137.0	121.3	131.7	131.6	128.5	105.4	138.9	105.8	118.7	68.0	116.4	124.6
Solyc08g016720	110.6	110.3	22.6	0.2	127.5	118.2	102.6	116.8	112.0	103.0	91.3	114.7	100.2	94.1	53.2	97.7	104.2
Solyc08g066650	92.4	90.9	24.0	0.3	94.7	96.7	86.8	94.8	94.9	88.6	68.2	90.3	79.2	77.5	42.4	82.8	83.0
Solyc08g066720	126.8	126.7	17.1	0.1	142.7	128.1	122.6	122.2	135.5	132.5	110.1	145.4	121.1	126.7	63.8	118.8	127.7
Solyc08g075490	104.9	104.5	23.1	0.2	105.1	109.9	104.1	94.9	105.2	101.8	76.9	104.5	85.4	87.0	51.2	91.6	98.5
Solyc08g081570	94.9	93.7	14.7	0.2	108.0	100.9	86.3	100.9	102.8	97.5	84.4	107.4	81.1	86.5	49.6	83.1	89.9
Solyc10g081650	137.1	136.0	19.9	0.1	161.0	140.8	130.7	143.0	151.1	145.0	129.7	167.7	122.0	126.3	69.4	126.9	133.2
Solyc11g011990	120.7	120.8	16.9	0.1	136.4	121.2	117.6	120.8	131.7	127.8	106.2	141.5	105.8	110.2	60.1	107.8	116.6
Solyc11g069380	93.9	93.5	19.4	0.2	103.7	95.4	88.0	93.6	98.2	89.3	75.6	94.1	80.4	81.5	46.8	84.8	86.3
Solyc12g098710	100.0	98.5	24.3	0.2	106.8	100.0	90.2	100.5	102.9	96.5	81.3	101.5	81.6	86.9	50.5	89.0	93.9
Solyc12g008980	112.9	114.1	17.4	0.2	142.4	119.0	103.3	133.5	124.4	118.9	114.1	134.3	99.0	98.7	54.1	106.7	106.5

Table S4. (continued)

Gene ID	E45	E49	E51	E54	E55	E57	E60	E64	E70	E71	E72	E76	E77	E82	E83	E88	E92
Solyc01g005940	89.2	112.0	121.1	107.0	122.7	115.9	100.5	128.9	110.1	120.7	118.1	102.9	136.2	96.4	121.6	104.1	112.7
Solyc01g009010	64.1	87.6	91.2	83.5	140.2	96.5	85.2	98.0	87.8	102.4	91.7	88.9	111.5	75.6	89.3	77.3	78.8
Solyc01g067890	84.7	107.1	112.8	107.1	184.2	129.8	115.9	128.7	116.3	137.4	123.4	122.5	150.6	98.4	112.9	100.6	101.3
Solyc01g087260	80.3	115.1	122.3	108.6	96.8	114.2	97.8	125.4	103.4	117.3	114.4	99.3	131.6	98.1	119.8	105.4	106.8
Solyc01g090660	80.3	114.1	124.3	110.6	172.8	125.8	108.6	130.2	111.5	132.1	121.1	114.7	144.9	102.4	121.5	105.7	112.8
Solyc01g097810	89.4	122.7	130.4	116.2	122.3	124.2	109.4	134.9	116.6	133.7	129.8	113.0	150.9	102.6	130.8	109.6	117.9
Solyc01g102820	90.4	112.6	118.9	110.0	193.3	120.3	104.4	128.3	112.1	130.8	123.1	109.4	140.0	97.3	119.7	104.0	105.1
Solyc01g109300	75.6	101.5	101.0	99.5	245.1	121.0	104.3	113.8	106.9	129.4	112.6	113.9	137.2	87.4	101.5	93.4	90.5
Solyc02g085700	110.7	153.8	160.7	143.1	158.9	155.2	126.2	169.1	144.7	162.4	163.6	135.4	173.3	134.5	157.1	143.4	134.1
Solyc02g090890	75.7	88.9	98.7	93.5	263.6	123.3	109.9	112.0	106.6	124.6	110.8	127.2	140.9	82.2	98.9	81.9	89.8
Solyc03g007960	80.4	106.4	106.2	104.0	165.4	118.6	101.5	120.2	108.0	128.0	112.4	111.9	137.9	180.5	109.1	96.7	94.4
Solyc03g031860	97.8	127.9	136.2	122.2	102.4	125.7	109.2	140.9	115.6	133.6	133.9	106.8	143.6	110.2	133.5	116.1	121.7
Solyc03g114340	91.3	104.4	117.1	112.7	231.4	138.6	125.0	131.7	121.9	142.3	126.7	135.3	164.8	100.0	125.0	97.4	109.4
Solyc03g123760	88.8	113.1	127.6	115.7	167.9	126.1	106.5	135.6	117.3	133.7	125.3	115.8	147.6	104.5	127.3	110.2	114.5
Solyc04g040190	83.8	102.9	105.5	95.7	148.7	109.3	105.4	121.6	107.5	122.3	114.7	113.6	132.0	89.4	107.1	94.9	100.4
Solyc04g050930	74.5	75.2	95.7	90.9	201.4	114.0	105.3	112.2	100.8	119.1	104.0	111.4	136.0	77.3	102.2	81.7	93.1
Solyc04g056390	140.4	180.4	188.6	164.8	153.8	168.0	152.1	205.9	158.9	187.4	177.4	146.2	209.3	145.5	187.8	166.0	184.2
Solyc04g079960	92.5	111.7	122.0	113.9	292.6	144.0	121.0	136.0	124.9	152.4	135.6	138.6	164.4	103.5	121.5	103.0	108.7
Solyc05g010180	75.5	93.5	99.2	89.4	128.5	98.9	91.8	108.1	88.0	107.8	99.3	89.5	119.2	77.0	100.3	83.2	98.9
Solyc05g016330	74.9	105.2	109.7	106.2	218.2	121.0	105.1	120.2	109.1	130.2	116.6	113.0	141.6	91.9	108.8	95.2	97.9
Solyc05g055760	85.9	113.9	118.4	108.3	187.6	122.9	104.2	129.2	109.4	128.1	123.3	109.5	135.2	97.2	111.8	104.3	99.5
Solyc06g036260	72.3	87.8	93.4	85.9	145.0	94.5	85.6	106.5	87.6	102.0	95.9	86.6	117.6	77.3	94.4	80.5	87.8
Solyc06g074240	128.2	174.6	192.0	161.5	126.8	173.0	142.1	200.0	160.9	163.2	178.3	152.4	199.5	150.1	187.9	160.4	161.7
Solyc07g056570	102.9	142.1	150.5	137.9	351.0	165.6	136.5	168.6	148.7	170.8	156.2	144.5	187.8	127.3	151.7	128.8	132.2
Solyc08g016720	92.0	114.4	120.0	115.0	209.8	119.0	115.1	136.5	115.3	139.7	126.6	113.7	158.6	99.7	122.5	100.9	116.4
Solyc08g066650	71.3	95.5	101.0	95.4	213.3	114.5	100.7	114.7	99.5	122.2	103.6	108.6	134.1	85.3	104.9	88.5	94.8
Solyc08g066720	102.5	148.6	143.9	133.4	130.7	141.2	123.9	155.4	128.4	149.5	143.8	123.9	156.4	125.0	141.8	135.8	126.4
Solyc08g075490	88.2	113.3	117.1	114.3	208.4	124.3	110.6	131.0	117.7	136.2	124.8	123.7	146.6	100.1	109.1	105.0	100.3
Solyc08g081570	75.6	104.7	113.7	97.7	124.7	104.9	87.7	118.3	98.1	111.5	108.0	91.1	130.5	90.9	114.8	95.5	106.7
Solyc10g081650	113.6	148.5	165.2	141.4	117.5	148.1	126.9	171.9	140.1	152.5	156.8	127.7	173.3	130.8	166.7	139.9	146.9
Solyc11g011990	104.9	135.0	134.8	123.4	144.7	134.3	114.2	150.7	123.5	140.9	139.9	115.7	151.2	116.8	136.3	122.9	122.3
Solyc11g069380	73.1	98.0	104.7	97.9	179.6	115.0	99.5	112.1	100.9	119.4	110.6	102.6	132.1	87.8	107.1	86.9	93.5
Solyc12g098710	77.7	106.6	114.9	104.0	226.0	120.6	101.5	125.1	106.0	126.1	116.2	106.1	138.5	94.6	110.9	98.3	97.9
Solyc12g008980	99.4	121.6	137.8	115.9	115.8	114.8	103.7	140.9	107.3	124.8	125.9	102.3	148.7	102.3	144.9	104.8	130.6

Table S4. (continued)

Gene ID	E93	E95	E98	E99	E102	E103	E105	E106	E108	E113	E115	E117	E118	E119	STR	URI	ZBR
Solyc01g005940	97.0	96.3	106.7	94.6	116.4	116.4	104.3	100.3	115.6	94.7	94.3	92.9	77.9	78.6	94.6	105.4	98.3
Solyc01g009010	71.0	78.1	81.6	70.9	83.0	83.0	83.6	75.1	86.2	76.6	70.5	71.6	54.7	57.7	70.9	81.3	78.4
Solyc01g067890	94.7	101.8	105.7	82.5	103.1	103.1	102.1	94.9	107.5	102.6	88.7	88.9	64.5	68.6	82.5	105.1	103.8
Solyc01g087260	93.9	96.3	101.3	96.6	118.4	118.4	107.6	100.1	115.4	94.6	87.2	88.6	76.5	75.4	96.6	99.3	98.3
Solyc01g090660	96.4	106.1	107.3	93.0	118.0	118.0	113.1	104.0	116.1	100.1	91.5	94.4	74.9	75.7	93.0	107.8	105.5
Solyc01g097810	105.2	104.3	114.4	101.1	124.6	124.6	117.6	108.0	123.2	104.6	93.6	94.8	78.8	79.9	101.1	110.9	111.4
Solyc01g102820	94.3	100.6	102.7	91.8	113.1	113.1	109.1	102.1	113.6	97.4	92.6	91.8	72.1	76.6	91.8	108.6	102.7
Solyc01g109300	81.7	97.1	92.0	80.5	100.7	100.7	99.3	88.5	102.5	94.4	77.5	85.2	64.2	67.9	80.5	100.1	96.2
Solyc02g085700	122.5	129.0	131.2	124.3	153.1	153.1	143.9	131.1	151.9	129.2	115.8	116.0	96.0	99.0	124.3	130.8	127.0
Solyc02g090890	80.6	87.6	96.4	69.3	86.8	86.8	85.1	81.5	89.2	91.6	75.4	74.6	56.8	56.3	69.3	88.5	69.5
Solyc03g007960	86.2	94.8	100.2	160.3	104.5	104.5	103.7	92.2	107.9	94.3	84.6	83.6	66.7	68.0	160.3	95.8	96.2
Solyc03g031860	108.0	104.0	118.3	110.7	129.9	129.9	118.5	114.3	128.6	104.9	103.2	105.7	87.8	89.0	110.7	121.7	110.8
Solyc03g114340	95.9	108.1	118.2	83.9	99.8	99.8	103.2	93.8	109.1	102.2	94.2	86.6	67.6	68.6	83.9	104.6	103.9
Solyc03g123760	101.6	104.1	112.6	93.1	116.1	116.1	111.8	103.2	118.9	102.5	94.8	89.6	76.8	75.6	93.1	103.6	106.3
Solyc04g040190	93.0	92.8	100.4	85.1	101.7	101.7	97.2	88.5	102.6	88.4	87.9	94.6	70.9	76.1	85.1	106.5	94.5
Solyc04g050930	85.9	86.9	101.0	69.0	86.8	86.8	84.3	74.6	86.6	85.2	77.8	74.3	59.5	58.6	69.0	92.3	85.9
Solyc04g056390	159.2	145.3	169.9	146.5	184.4	184.4	158.8	149.9	175.7	139.8	152.2	148.9	122.5	126.4	146.5	173.1	157.8
Solyc04g079960	100.9	109.0	116.2	85.6	111.1	111.1	106.3	100.6	116.4	107.5	93.0	84.4	69.6	73.3	85.6	105.2	107.7
Solyc05g010180	88.3	82.3	95.9	79.0	98.9	98.9	87.0	83.0	91.0	79.1	80.3	80.0	69.9	70.4	79.0	95.9	79.9
Solyc05g016330	87.2	97.5	97.0	82.2	102.1	102.1	97.6	92.0	106.7	98.5	82.8	82.7	64.1	68.7	82.2	96.7	96.7
Solyc05g055760	89.4	101.0	95.2	91.3	113.4	113.4	111.2	100.5	113.8	101.0	88.3	93.7	73.4	77.4	91.3	105.1	102.8
Solyc06g036260	78.5	78.6	92.0	73.5	92.8	92.8	83.7	76.4	90.6	76.4	77.2	76.2	63.0	63.5	73.5	89.2	58.0
Solyc06g074240	151.3	144.3	161.0	148.2	181.2	181.2	159.8	152.2	179.8	138.5	141.2	139.2	117.9	119.4	148.2	155.2	152.9
Solyc07g056570	117.4	128.5	131.2	105.8	132.1	132.1	132.1	117.6	138.7	129.2	111.5	104.3	85.1	90.0	105.8	122.3	130.8
Solyc08g016720	102.4	102.3	114.5	87.8	119.0	119.0	103.9	98.2	110.1	100.2	101.7	104.3	79.0	82.7	87.8	126.8	110.3
Solyc08g066650	82.9	89.0	91.1	74.3	94.3	94.3	90.9	80.8	95.8	89.8	77.4	75.7	60.2	62.8	74.3	92.5	88.9
Solyc08g066720	111.3	107.0	119.4	120.5	138.6	138.6	136.3	124.3	142.9	116.8	108.8	118.6	93.3	97.9	120.5	134.8	122.2
Solyc08g075490	91.2	101.1	104.2	88.6	108.9	108.9	106.3	100.6	115.5	104.7	88.0	89.7	68.3	72.6	88.6	100.9	107.2
Solyc08g081570	89.7	90.4	95.7	82.3	102.0	102.0	92.5	88.7	100.4	86.4	83.1	81.8	70.6	70.0	82.3	93.7	93.6
Solyc10g081650	128.5	125.4	136.0	127.4	158.0	158.0	140.2	133.3	157.7	124.9	118.5	117.6	103.6	101.8	127.4	133.9	136.4
Solyc11g011990	111.2	112.2	121.3	110.9	137.2	137.2	125.0	118.8	133.5	113.2	102.4	105.1	89.2	91.6	110.9	119.1	119.4
Solyc11g069380	84.3	91.8	95.0	79.0	95.9	95.9	92.0	89.2	102.8	90.8	80.8	80.2	66.6	64.6	79.0	95.5	89.4
Solyc12g098710	87.6	96.2	99.5	83.6	102.8	102.8	98.5	89.4	104.3	95.6	86.7	84.5	68.6	69.2	83.6	98.8	97.0
Solyc12g008980	115.2	98.8	122.3	101.3	121.7	121.7	108.0	102.8	124.0	101.0	101.9	94.6	93.6	82.4	101.3	114.6	106.9

Table S5. List of non-redundant polymorphisms detected across all 47 genotypes classified as high-, medium- and low-carotenoid content (CC). (as separate file)

Table S6. List of the 671 non-redundant mutation events affecting gene regions in 30 out of 34 candidate genes. (as separate file)

Table S7. List of genes and specific primer pairs flanking SNP/InDel sites designed for PCR amplifications prior to Sanger sequencing.

Gene ID	Enzyme	Primer ID	Primer name	Primer sequence	Tm (°C)	Amplicon bp	Genomic coordinate on SL2.50	Reference allele	Alternative allele	Type	Genotype	PCR	Sequencing	SNP/InDel validation
Solyc01g090660	**CCD7**	**14**	CCD7_F<br>CCD7_R	TTTTGCTTGTAAGCCTGACATC<br>CTGTCATTGATCCTGCAACG	68<br>68	574	84309670	A	G	Missense	E1	+	–	
Solyc01g097810	**ZDS**	**15**	ZDS_F<br>ZDS_R	CCATCCAAACATGCCATAAA<br>ACAGATCAGGTTGAAATGACCTC	64<br>70	797	88515979	A	G	Missense	STR	+	+	+
Solyc01g102820	**MCT**	**6**	MCT_F<br>MCT_R	GCACGCTTTTGTCAAAGTCTC<br>CATCACCCGCCAGTTAAGTT	63<br>68	867	91518898	A	T	Stop lost	STR	+	+	+
Solyc02g085700	**GGPPS3**	**1**	GGPPS3_F<br>GGPPS3_R	TGCAGTTGCGTCTCTTCACT<br>AATTCTTTTCCCACCAGCAA	68<br>64	483	43100279	C	CTTGTAGCA	Intergenic	ZBR	–		
		**2**	GGPPS3_F1<br>GGPPS3_R1	CCCTTTTTCTTAGTTGGCATGA<br>TCTTCGATCGAGTTAATTCCCTA	68<br>68	592	43099996	A	ATAT	Intergenic	ZBR	+	+	+
Solyc03g031860	**PSY1**	**7**	PSY1_F<br>PSY1_R	TGGTGAAGAGGCAACTGAGA<br>GAACAGCAACGCAAATGAAA	68<br>64	541	4327099	T	C	Splice donor	E40	+	+	+
		**8**	PSY1_F1<br>PSY1_R1	GGAAAAGTTGGTTTGCCTGT<br>TCTCAGTTGCCTCTTCACCA	66<br>68	502	4326668	C	T	Stop gained	E93	–		
		**9**	PSY1_F2<br>PSY1_R2	TTTTGCTTGTAAGCCTGACATC<br>CTGTCATTGATCCTGCAACG	68<br>68	574	4326829<br>4326829	G<br>G	GCCGCACAACA<br>T	Frameshift<br>Missense	E93	+	–	
Solyc03g114340	**DXR**	**3**	DXR_F<br>DXR_R	CCACTAAAAAGACACGGAATGA<br>TCTTTAACCACCCCCACAAC	68<br>68	637	58400626<br>58400636<br>58400657<br>58400673	G<br>C<br>A<br>CTT	A<br>T<br>T<br>C	Intergenic<br>Intergenic<br>Intergenic<br>Intergenic	STR	+	–*<br>+<br>+<br>–*	 +<br>+
Solyc04g056390	**IPP1**	**10**	IPP1_F<br>IPP1_R	AATCCAAACGCAGCTCAAAT<br>AGCCCCATTTCCTTTACCAC	64<br>68	528	54167730	T	A	Splice acceptor	BX	+	+	+
Solyc05g016330	**CYP97B2**	**16**	CYP97B2_F<br>CYP97B2_R	TCAATCGATCAAATTGGGAAC<br>AGCTTCATCCCTGTGAATGG	65<br>68	604	15575163<br>15575186	T<br>C	A<br>T	Missense<br>Missense	E16	+	+<br>+	+<br>+
Solyc06g036260	**CHY1**	**17**	CHY1_F<br>CHY1_R	CATCAAAAAGAGGCAACTGG<br>CGGTTTGGGTCGTGACATA	66<br>68	496	25744263	T	A	Missense	ZBR	–		
Solyc06g074240	**CYCB**	**11**	CYCB_F<br>CYCB_R	GGAGAGTGGTGAAGGGTCAA<br>GGGATTGGGAAAAGGGTAAA	70<br>66	588	45899612	AT	A	Frameshift	ZBR	+	+	+
		**12**	CYCB_F1<br>CYCB_R1	TATCCCTGAATTCCCACCAA<br>TTGACCCTTCACCACTTTCC	66<br>68	746	45898857	T	A	Missense	E71	+	+	+
Solyc08g016720	**NCED2**	**18**	NCED2_F<br>NCED2_R	TCAATGAATGTAATGAGAATTTAAAGA<br>CCGATAATTTTCGTGGTATTATACTTG	66<br>70	506	8736005	C	G	Missense	E88	+	–	
Solyc08g066650	**CCD8**	**13**	CCD8_F<br>CCD8_R	AGTCCATGCAGCAAGCTTTT<br>AATTTTGAATCCGCCATCTG	66<br>64	430	55414050	GGTT	G	Inframe deletion	STR	+	–	
Solyc08g066720	**CCD_like**	**19**	CCD_like_F<br>CCD_like_R	TGCAGAGCTCGTGAATCAGT<br>TGGCAAGCCCTCCATATTTA	68<br>66	403	55543988	T	A	Missense	STR	+	+	+
Solyc10g081650	**CtrIso**	**4**	CtrIso_F<br>CtrIso_R	CAAAAGAAGCGCCAAACAAT<br>TCAACTCAACCCAACACCAA	64<br>66	529	62012898	T	TACTTTGAAGGAACCC	Intergenic	STR	–		
		**20**	CtrISO_F<br>CtrISO_R	GGCCAATGAGGAATCAAGAA<br>GGAGAACGAAGAGGGAAGAA	66<br>68	853	62684196	A	G	Missense	ZBR	–		
Solyc11g069380	**HDS**	**5**	HDS_F<br>HDS_R	TCCAATGAAAAGCAAACACG<br>GGATACCACCTAGGGCAAAA	64<br>48	724	51061535<br>51061576<br>51061579	A<br>G<br>A	AGTAATAATTCATT<br>A<br>G	Intergenic<br>Intergenic<br>Intergenic	E54	+	+<br>+<br>+	+<br>+<br>+

* Low quality sequencing

48

Supplementary Figures

Figure S1. Selecting the optimal number of groups (K) with the Elbow method.

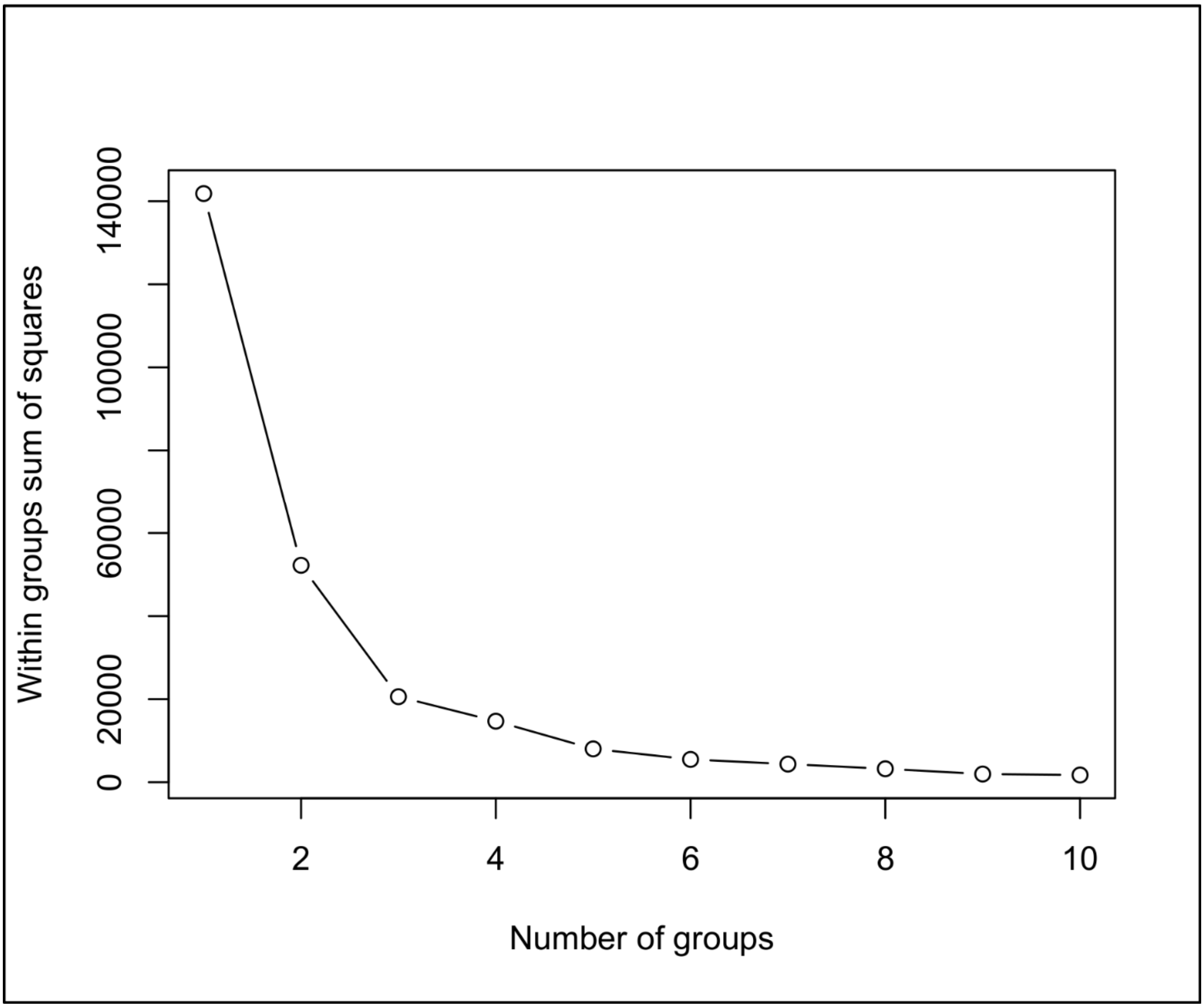

Figure S2. Venn diagram showing the number of sequence changes restricted to genotypes within the low-, medium- and high-CC classes.

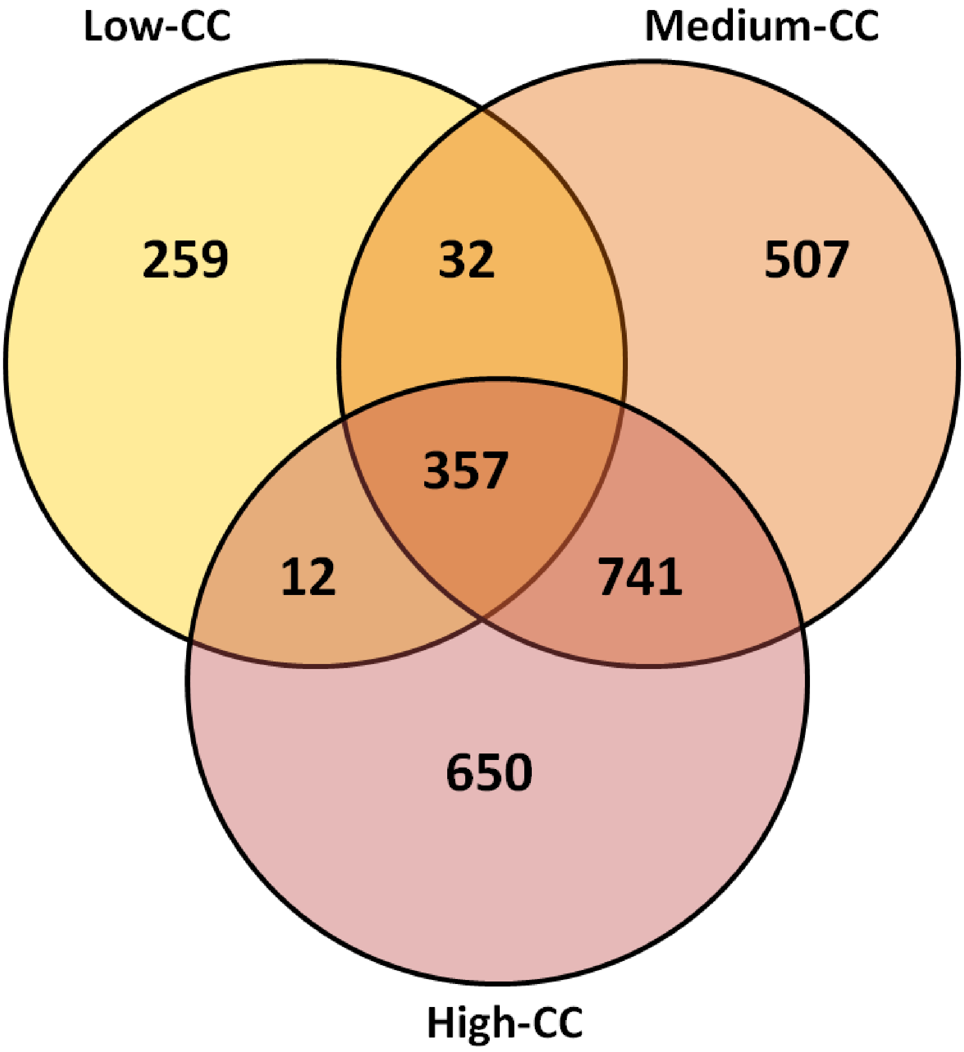

Figure S3. Gene structures and genomic coordinates of SNP/InDel polymorphisms across 30 tomato genes. Black boxes and strand lines represent CDS exons and introns, respectively. Red boxes represent UTRs. (as separate file)

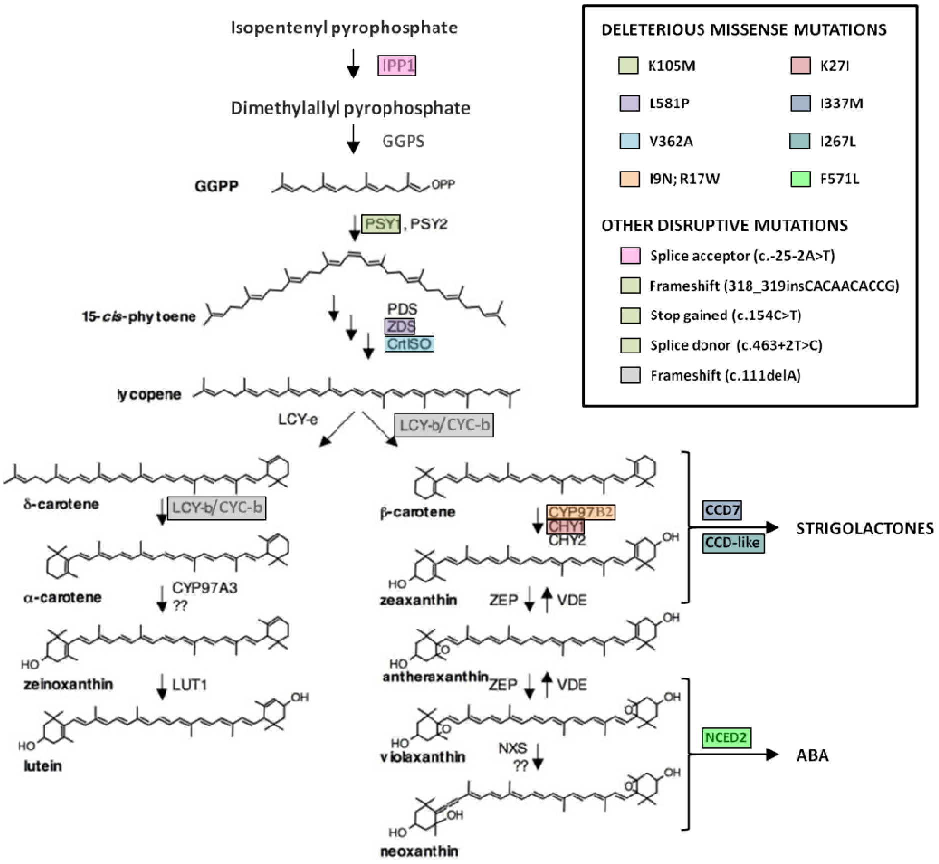

Figure S4. Schematic representation of the main steps of the MEP carotenoid pathway. Genes affected by 9 missense deleterious mutations as well as by other disruptive mutations are highlighted. Modified from Diretto *et al.*, 2006[62].

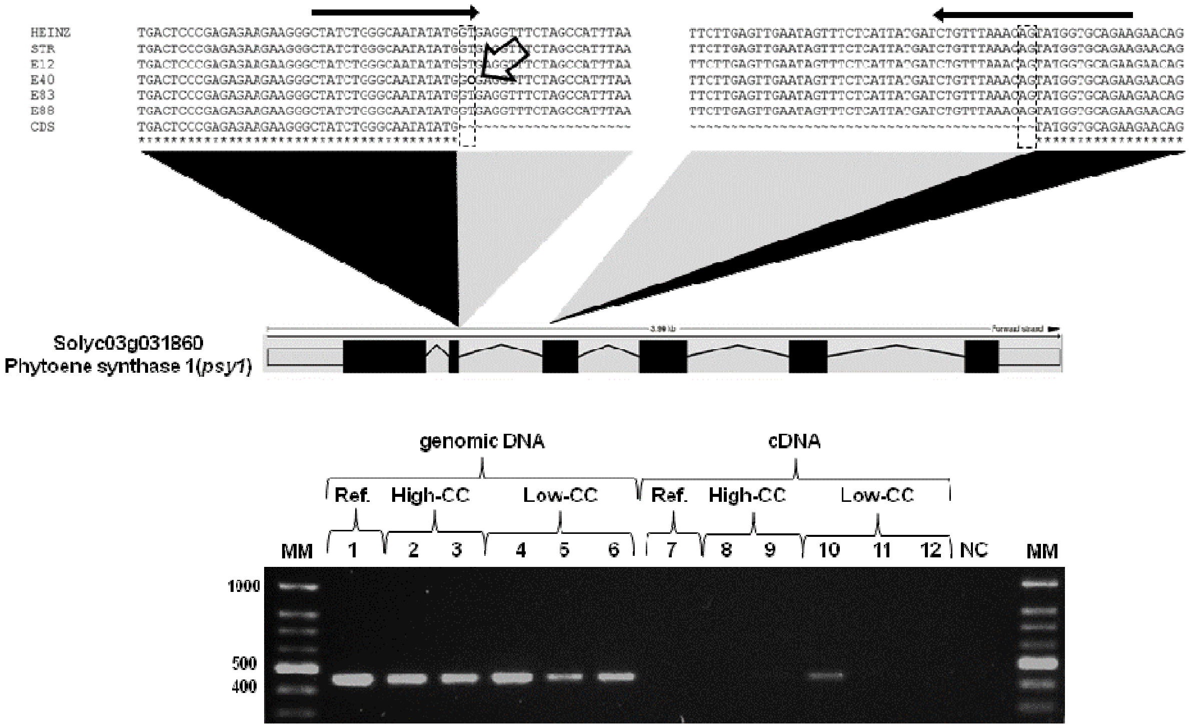

Figure S5. PCR results on the detection of the intron II retention event of the phytoene synthase 1 (*psy1*) gene. From top to bottom:

Multiple sequence alignment (MSA) of the mRNA region ranging from the end of exon II to the beginning of exon III for six tomato genotypes, namely Heinz 1706 (Ref.); STR and E12 (belonging to the high-CC class); E40, E83 and E88 (belonging to the low-CC class). The CDS of Heinz 1706 is included in the MSA so that intron II splice sites can be highlighted. Forward and reverse primers (black arrows with reverse orientation) target splice donor and splice acceptor sites (dashed boxes), respectively. The hollow arrow points to the T→C point mutation in the splice donor site of exon-intron junction II which affects the E40 genotype.

Schematic representation of the *psy1* gene structure. Black boxes and strand lines represent CDS exons and introns, respectively. Empty boxes represent UTRs.

Cropped gel showing electrophoresis pattern of PCR products for detection of the intron retention event. Full-length gel is presented in Supplementary Figure S6. Genomic DNA and mRNA were isolated from leaves and then were subjected to PCR analysis. PCR amplification results on genomic DNA (lanes 1-6) and cDNA (lanes 7-12) are reported for the six genotypes listed above (same order). PCR product of ~450bp in size in lane 10 confirms the retention of the second intron in the genotype E40. MM: molecular markers, NC: negative control.

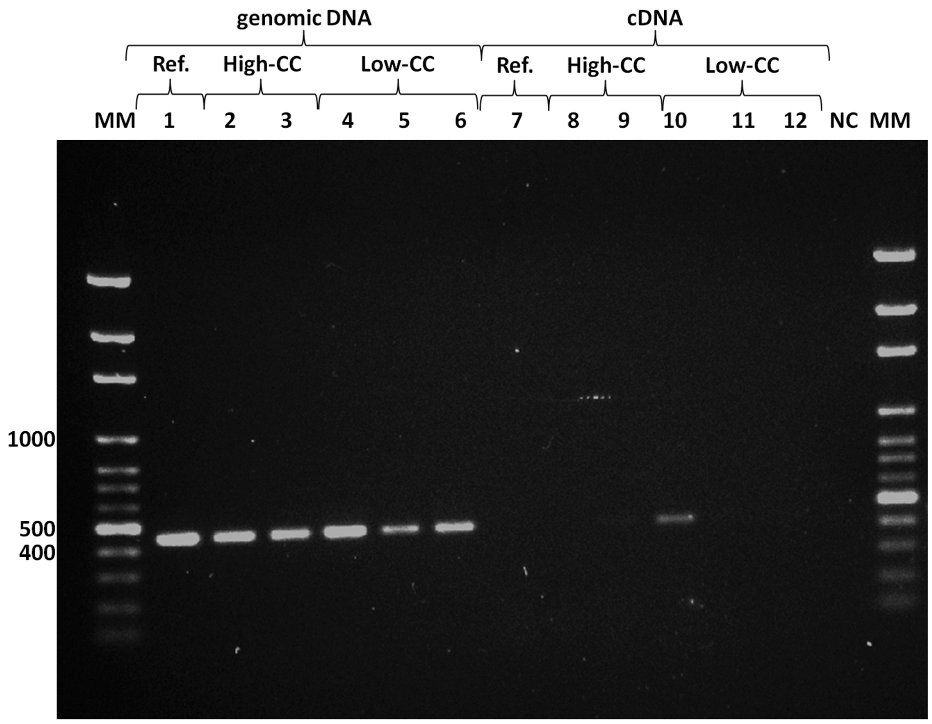

Figure S6. Full-length gel displayed in Figure S5. Genomic DNA and mRNA were isolated from leaves and then were subjected to PCR analysis. PCR amplification results on genomic DNA (lanes 1-6) and cDNA (lanes 7-12) are reported for the six genotypes: lanes1 and 7: Heinz 1706 (Ref.); lanes 2 and 8: STR; lanes 3 and 9: E12; lanes 4 and10: E40; lanes 5 and 11: E83; lanes 6 and 12: E88; MM: molecular markers, NC: negative control. High-CC: high carotenoid content; Low-CC: low carotenid content.

Supplementary Files

File S1. For each candidate gene, it is reported the list of *cis*-acting regulatory elements present within the promoter region and their copy number across all 47 genotypes as detected by PLACE db analysis. (as separate file)
