# Supplementary figures and images for: Liquid-phase sequence capture and targeted re-sequencing revealed novel polymorphisms in tomato genes belonging to the MEP carotenoid pathway
Source: Sci Rep. 2017 Jul 17;7:5616. doi: 10.1038/s41598-017-06120-3 (PMC5514110; doi:10.1038/s41598-017-06120-3)

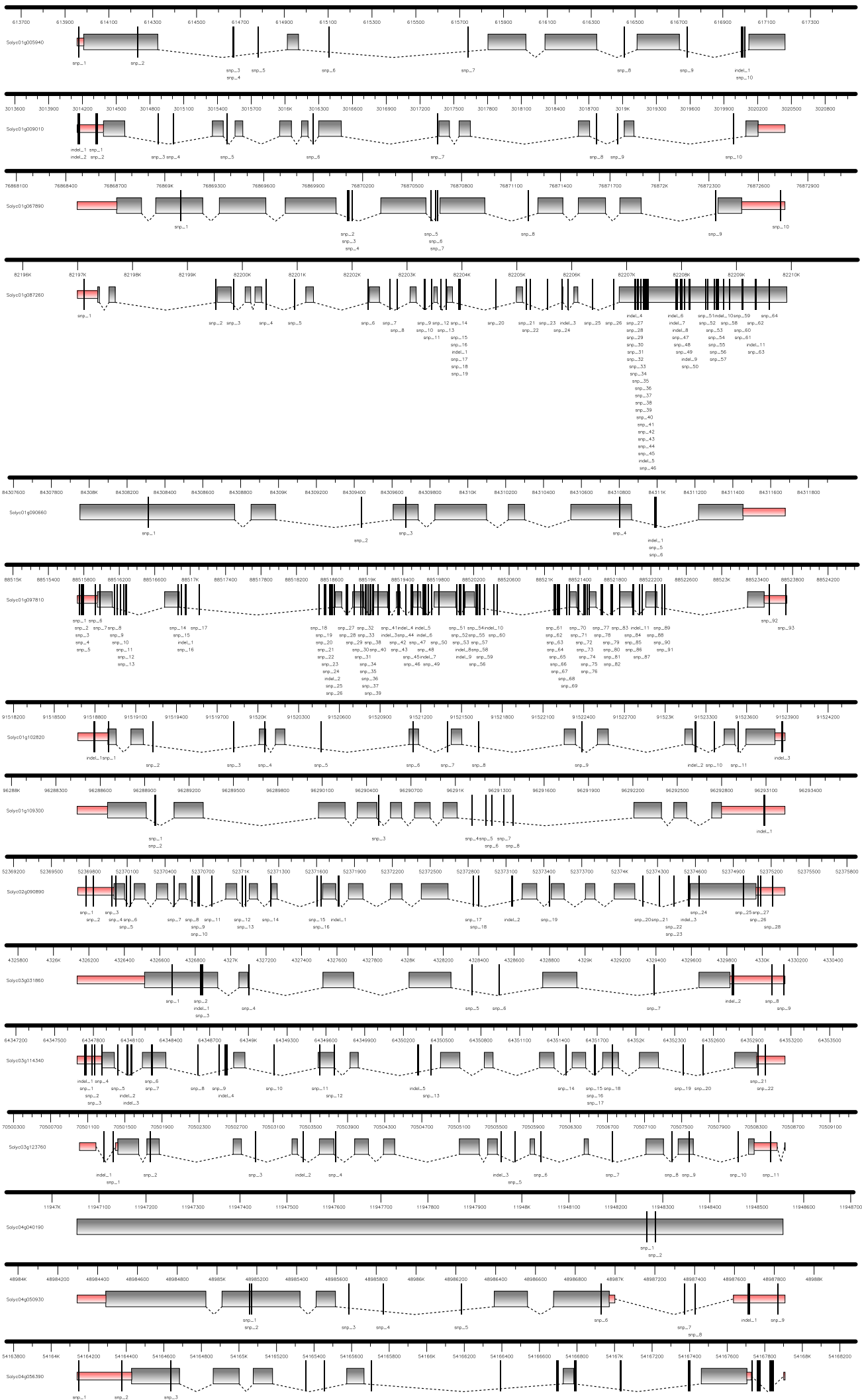

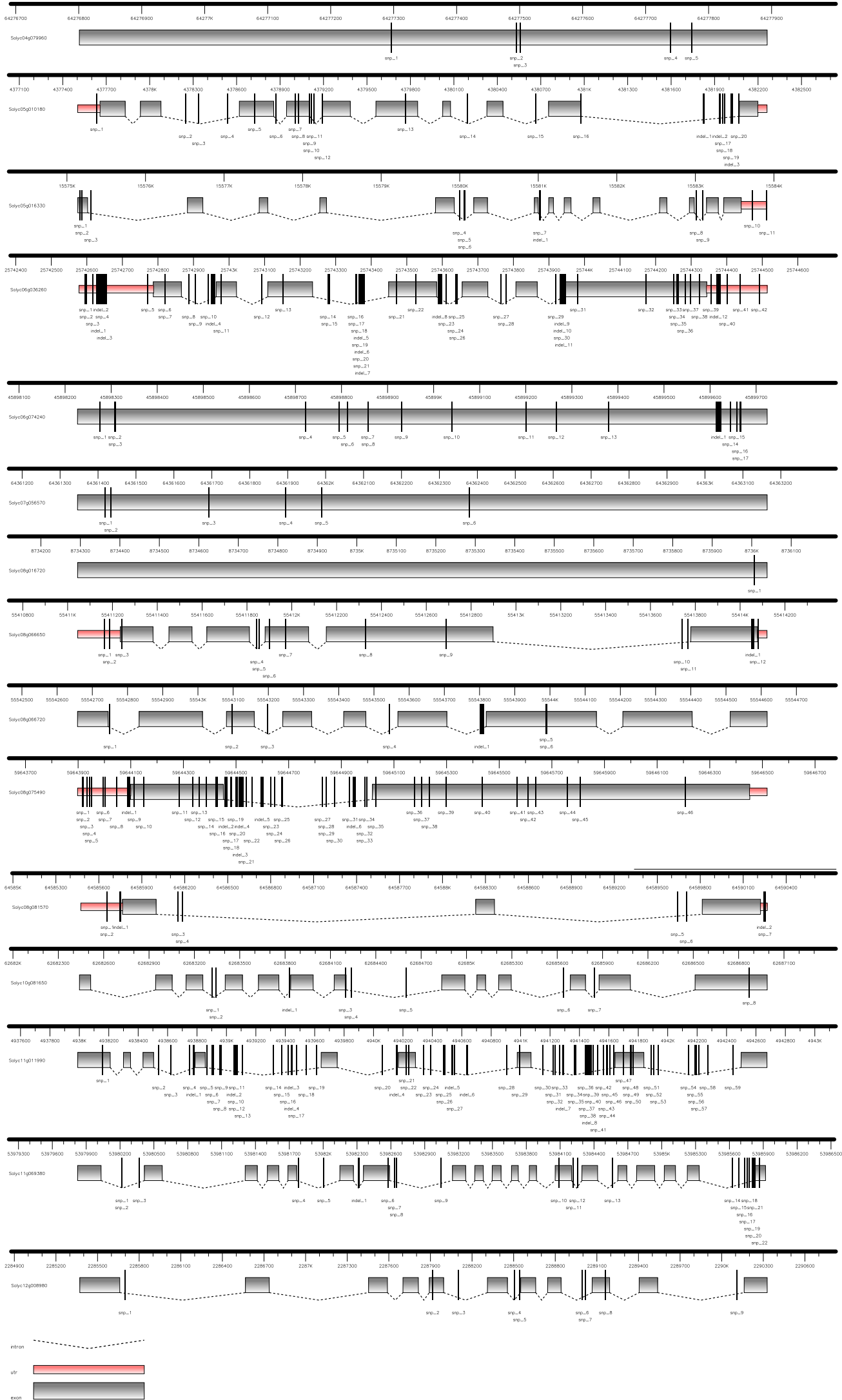

Supplement: Supplementary file 2 — Figure S3 [file 41598_2017_6120_MOESM2_ESM.pdf]
